# Effects of temperature, chloride and perchlorate salt concentration on the metabolic activity of *Deinococcus radiodurans*

**DOI:** 10.1007/s00792-024-01351-5

**Published:** 2024-07-24

**Authors:** Eftychia Symeonidou, Uffe Gråe Jørgensen, Morten Bo Madsen, Anders Priemé

**Affiliations:** 1 Astrophysics and Planetary Science, Niels Bohr Institute, University of Copenhagen, Øster Voldgade 5-7, 1350 Copenhagen, Denmark; 2 Center for ExoLife Sciences, (CELS), Niels Bohr Institute, University of Copenhagen, Øster Voldgade 5-7, 1350 Copenhagen, Denmark; 3 Department of Biology, University of Copenhagen, Universitetsparken 15, 2100 Copenhagen, Denmark

**Keywords:** Martian regolith, Metabolic Activity, Viability, Halotolerant, Psychrotolerant

## Abstract

The extremophile bacterium *Deinococcus radiodurans* is characterized by its ability to survive and sustain its activity at high levels of radiation and is considered an organism that might survive in extraterrestrial environments. In the present work, we studied the combined effects of temperature and chlorine-containing salts, with focus on perchlorate salts which have been detected at high concentrations in Martian regolith, on *D. radiodura*ns activity (CO_2_ production rates) and viability after incubation in liquid cultures for up to 30 days. Reduced CO_2_ production capacity and viability was observed at high perchlorate concentrations (up to 10% w/v) during incubation at 0 or 25 °C. Both the metabolic activity and viability were reduced as the perchlorate and chloride salt concentration increased and temperature decreased, and an interactive effect of temperature and salt concentration on the metabolic activity was found. These results indicate the ability of *D. radiodurans* to remain metabolically active and survive in low temperature environments rich in perchlorate.

**Supplementary Information:**

The online version contains supplementary material available at 10.1007/s00792-024-01351-5.

## Introduction

Earth is a mosaic of different habitats, some of which provide extremely harsh living conditions for most organisms. However, extremophiles have adapted to a variety of adverse conditions thus expanding the limits of life. One of the main quests of Astrobiology is to explore whether such organisms are capable to survive and sustain activity outside our planet. The most prominent and well-studied candidate for testing this aim is our solar neighbour planet, Mars.

The conditions prevailing on the Martian surface, nevertheless, make it a multi-extreme environment that poses several challenges for extremophiles. The adversities on Mars include low temperatures that vary between 180–280 K (−93 to 7 °C) (Kieffer 1976; Smith et al. 2004), high amounts of both UV and cosmic radiation (Cockell et al. 2000; Hassler et al. 2013), lack of water, i.e. liquid H_2_O (Catling 2014), and a thin atmosphere consisting mainly of CO_2_, N_2_ and Ar (Catling 2009).

Several past missions to Mars have studied the geochemistry of the planet with one of the most interesting findings being the detection of chlorine (Hecht et al. 2009). Focus has been given to the perchlorate anion (ClO_4_^−^) which has been found at 0.5–0.7 wt%, mainly coupled with calcium or magnesium in the form of salts (Ca(ClO_4_)_2_ and Mg(ClO_4_)_2_) (Kounaves et al. 2014). These salts can be hazardous especially to human health (Niziński et al. 2020) but they are also highly deliquescent and can lower the freezing temperature of water, forming liquid brines that could potentially host life (Martínez and Renno 2013).

The survival of any earthly organism under these conditions would be a great challenge evidently. One of the suggested solutions to this has been the creation of underground habitats in the case of colonization (Liu et al. 2022), though some extremities such as radiation (Röstel et al. 2020), perchlorates (Martín-Torres et al. 2015) and low temperatures might still exist in the subsurface. Consequently, organisms capable to survive and thrive in such conditions would be needed for the first steps of Martian colonization.

The bacterium *Deinococcus radiodurans* is considered as one of the most notable extremophile species due to its ability to survive under extreme ionizing radiation (Cox and Battista 2005). Different previous studies have investigated the ability of *D. radiodurans* to survive under different temperatures and the presence of salts. More specifically, Airo et al. (2004) found that cold-tolerance was increased after incubation at 20 °C followed by consecutive cold-shock cycles, while survival at -35 °C was found after incubation in soil soaked with saline water or seawater (Diaz and Schulze-Makuch 2006). These properties of *Deinococcus radiodurans* render it already an important candidate for Mars colonization plans.

Even though the survival of *D. radiodurans* at different irradiation and temperature conditions has been investigated, its ability to retain its metabolic activity in perchlorate-rich environments has not been thoroughly studied. As temperature exerts great influence on the metabolism of most microorganisms, the current work focused on the combined effects of temperature and calcium or magnesium perchlorate salts on the ability of *D. radiodurans* to remain metabolically active. To understand if *D. radiodurans* is sensitive to high osmolarity or to perchlorate salts specifically, we also tested it with chloride salts separately. We hypothesized that *D. radiodurans* would remain metabolically active at the lower salt concentrations and at a medium range temperature that is near-optimal for its growth. We also hypothesized a reduction of activity as the concentration of salts, especially perchlorate ones, is increased and the temperature is lowered. In addition, we tested the *viability* of *D. radiodurans* cells under the same hypotheses and after long-term exposure to the same incubation temperature, salts and concentrations as referred above.

## Materials and methods

### Bacterial cultures and growth conditions

*Deinococcus radiodurans* strain DSMZ 20539 was obtained from the German Collection of Microorganisms and Cell Cultures. The initial growth conditions were incubation at 25 °C on Corynebacterium agar plates (DSMZ Medium 53). Subsequently, bacterial single-cell colonies were inoculated and incubated in liquid medium of the same composition without the presence of agar.

**Fig. 1:**
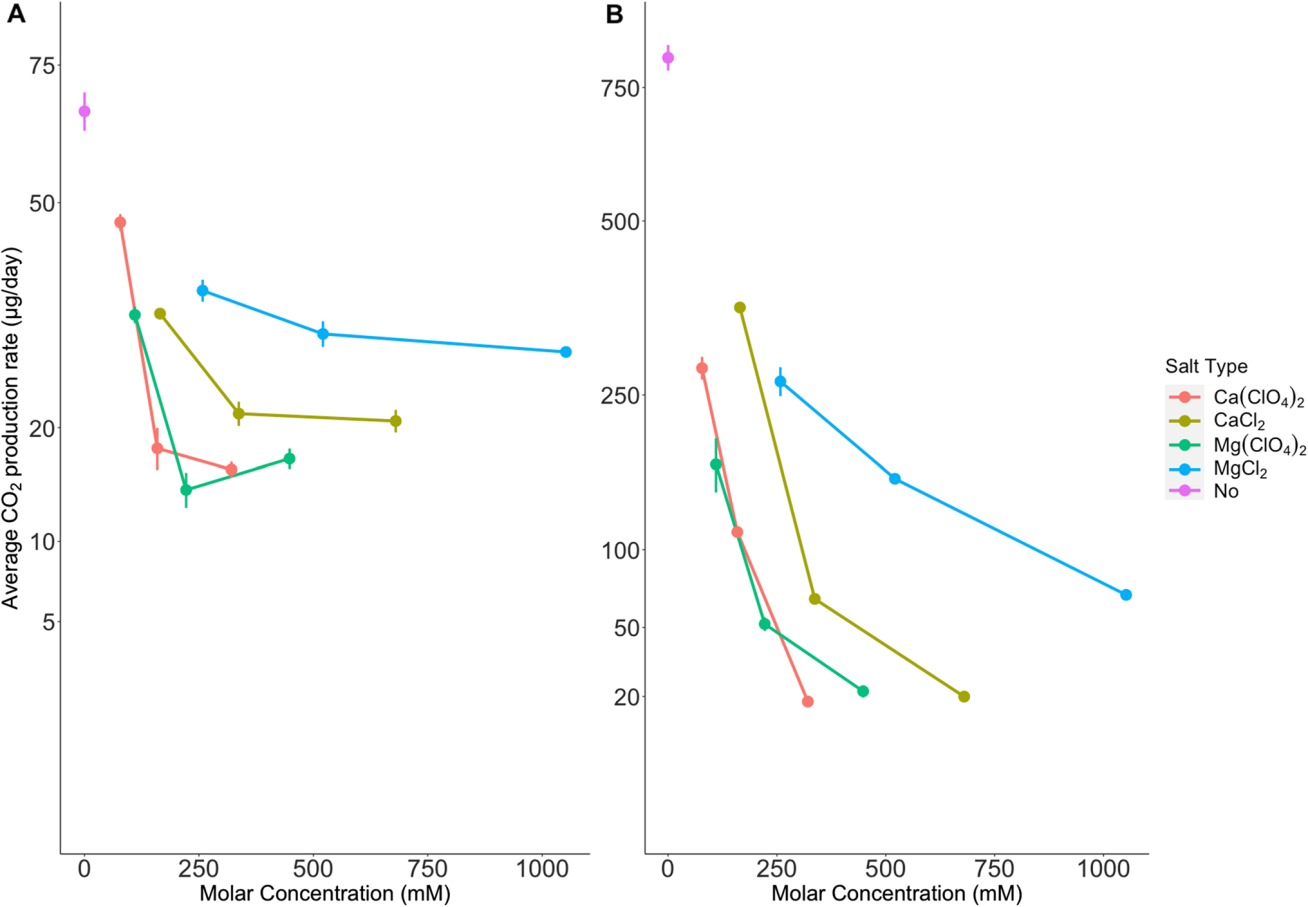
*Deinococcus radiodurans* CO_2_ production rates when incubated at different concentrations of perchlorate and chloride salts (magnesium or calcium for both cases) at two different temperatures **A** 0 °C and **B** 25 °C. Each dot represents the average CO_2_ production rate for each treatment and the error bars represent the standard error from the average. The rates were calculated based on the average CO_2_ production until the 20th day of the experiment. The y-axis is plotted on square root scaling in both subfigures and the scale of y-axis differs by an order of magnitude

Before the start of the experiment, the growth of *Deinococcus radiodurans* was assessed using growth curves. The bacteria were grown with shaking at 40 rpm. Growth curves were produced using both optical density measurements and CFU counts (Supplementary File 1, Sect. “Introduction”, Tables 1 & 2 and Fig. 1). The bacteria were also observed under light microscope during this phase.

All media and equipment used were autoclaved at 121 °C before use.

### Experimental setup

The media described above were mixed with four different salts at four different concentrations. The four salt treatments were *calcium chloride* (CaCl_2_·2H_2_O*,* Sigma Aldrich), anhydrous *magnesium chloride* (MgCl_2_, Sigma Aldrich), *calcium perchlorate (Ca(ClO*_*4*_*)*_*2*_·4H_2_O, Thermo Scientific) and anhydrous *magnesium perchlorate* (Mg(ClO_4_)_2_, Thermo Scientific). The weight and molar concentrations are found in Table 1.

**Table 1:** | Concentration and amount of salts used in the experiments. The weight and the amount of each salt were calculated for the final volume of liquid in each jar after the addition of liquid culture. Further information in Supplementary File 1, Sect. “Introduction”, Table 4

Salt concentration (%w/v)				Amount of salt (mM)			
CaCl_2_	MgCl_2_	Ca(ClO_4_)_2_	Mg(ClO_4_)_2_	CaCl_2_	MgCl_2_	Ca(ClO_4_)_2_	Mg(ClO_4_)_2_
0	0	0	0	0	0	0	0
1.8	2.5	1.9	2.5	165	258	78	110
3.7	5	3.8	5	337	521	159	222
7.5	10	7.7	10	680	1052	321	448

Each incubation jar contained only one salt type. The salt in each case and concentration treatment was homogenously mixed with the liquid growth medium at a final volume of 9 mL and the mix was added in 98-mL glass jars sealed with a butyl rubber stopper. Afterwards the sealed jars containing the salt-medium mix were autoclaved at 121 °C.

For each concentration of salt, six jars in total were prepared. For the study of the metabolic activity of *D.radiodurans*, three jars of each treatment (technical replicates) were incubated with the bacteria. Before initiation of the experiment, the liquid precultures were examined by optical microscopy to assess any contamination. Afterwards, the optical density of the cultures was determined using spectrophotometry at 600 nm (OD_600_). The optical densities for each preculture can be found in Supplementary section (Supplementary File 1, Sect. “Introduction”, Table 3). Finally, 1 mL of preculture was added with sterile syringe to the jars, with the final volume being 10 mL.

All cultures were incubated at 0 or 25 °C for 30 days in total.

During the incubation period, 3 mL headspace samples were obtained at specific time intervals and stored in 3 mL pre-evacuated Exetainer vials (*Labco Ltd, Lampeter, UK*). The CO_2_ concentration in each vial was measured with gas chromatography (*Thermal Conductivity Detector and SRI310C GC (SRI Instruments, Torrance, CA, USA)*). CO_2_ production rates were obtained from linear regression analysis of the increase in CO_2_ concentration over time.

The rest of the jars prepared for each treatment type (technical replicates) were prepared and incubated with the exact same procedure as described above in order to assess the number of surviving bacteria using CFU (Colony Forming Units) counts. On Day 5, 10 and 30 of the experiment, 10 μL culture was diluted in different fractions (Supplementary File 1, Sect. “Materials and methods”, Tables 5–14) with the dilution growth medium containing no perchlorate/chloride followed by culturing on agar plates containing no perchlorate/chloride. The plates were incubated at 25℃ for three to five days and the number of colonies on each plate was counted. Plates for CFU counts were also prepared from the precultures used in the experiment before they were added to the incubation jars.

None of the cultures used either for gas selection or CFU counts were incubated under shaking conditions.

## Results

### CO_2_ production rates

**Fig. 2:**
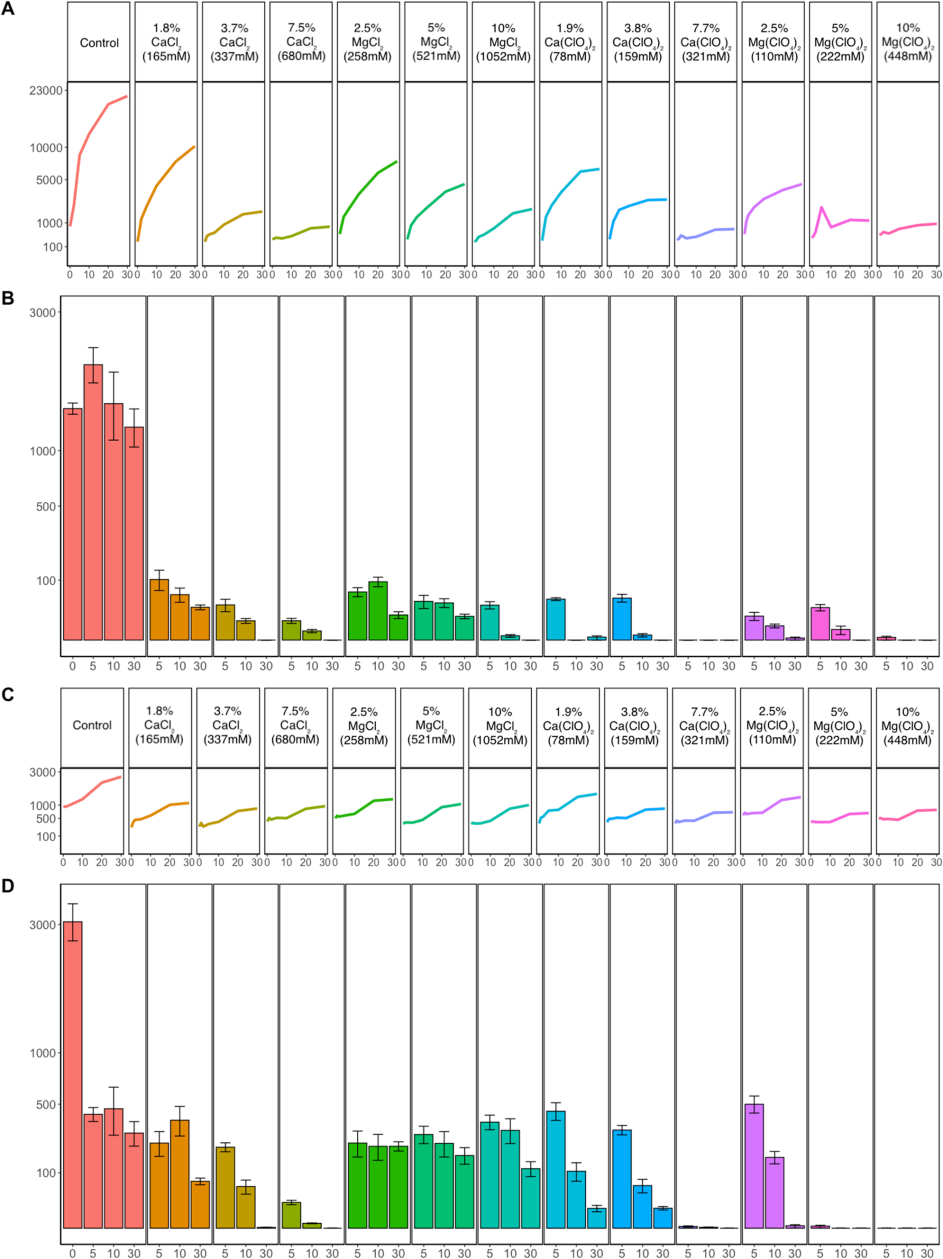
*Deinococcus radiodurans* average CO_2_ production and CFUs produced for the 30 days of the experiment following incubation at different concentrations of perchlorate and chloride salts (magnesium or calcium for both cases) at two different temperatures. **A** Average CO_2_ production per treatment culture at 25 °C. **B** Average CFUs produced per treatment culture at 25 °C. **C** Average CO_2_ production per treatment culture at 0 °C. **D** Average CFUs produced per treatment culture at 0 °C. All y axis are plotted with square root scaling. In figures A and C the y-axis scale limits differ. In figures B and C the bars represent the mean number of bacterial cells per ml of culture and the error bars represent the standard error of the mean

The ability of *D. radiodurans* to sustain activity at high perchlorate and chloride salt concentrations was tested during incubation at two different temperatures (0 and 25 °C). The CO_2_ production of each culture was measured at different time points during the 30-day incubation period (Supplementary File 1, Sect. “Discussion”, Figs. 2–9 & Supplementary File 2 & 3).

The average CO_2_ production rates were calculated from the results acquired over the first 20 days of the experiment as a plateau in CO_2_ concentration was observed between 20 and 30 days of incubation in certain cases (see Supplementary File 1, Sect. “Discussion”, Figs. 2–9). The acquired average CO_2_ rates for the same salt treatment were greater at 25 °C than 0 °C (Fig. 1 and Supplementary Files 2 & 3). The only exception can be found in the cultures treated with 7.5% w/v of CaCl_2_ (680 mM CaCl_2_) where the production rate was slightly larger for those growing at 0 °C. The largest difference was observed for the control culture where the rates were ~ 12 times greater at 25 °C (Fig. 1B) than at 0 °C (Fig. 1A).

The average CO_2_ production rates decreased with increasing salt concentration, except for Mg(ClO_4_)_2_ at 0 °C where the rates were slightly greater at 10% w/v (448 mM) compared to 5% w/v (222 mM) (Fig. 1A). The overall results presented in Fig. 1 show that *D. radiodurans* has consistently higher CO_2_ production rates when incubated at high concentrations of chloride salts compared to perchlorate salt, and this is consistent at both incubation temperatures.

For identically treated cultures, we calculated the ratio of CO_2_ production rate at the two incubation temperatures. This ratio decreased as the concentration of salt increased (Table 2) indicating that the effect of temperature decreases as the salt concentration increases. This result is supported by running a one-way ANOVA test (p-value = 3*10^–4^, see Supplementary File 1, Sect. “Results”).

**Table 2:** | Average CO_2_ production per treatment and temperature and the ratio resulting from the division of the rates for the same treatment at the two temperatures. The average CO_2_ production is calculated as the mean of CO_2_ per technical replicate at a specific treatment and temperature (3 replicates in each case). The value of the numerator (A) is the mean of CO_2_ at 25 °C and the mean of 0 °C is the value of the denominator (B). Further information on Supplementary File 1, Sect. “Discussion”, Figs. 2–9 and Supplementary Files 2 and 3

Treatment	Average CO_2_ production at 25 °C (μg/day)	Average CO_2_ production at 0 °C (μg/day)	Ratio (A/B)
Control	812.63	66.03	12.31
1.8% CaCl_2_ (165 mM)	364.91	33.52	10.89
3.7% CaCl_2_ (337 mM)	66.35	21.48	3.09
7.5% CaCl_2_ (680 mM)	19.96	20.7	0.96
2.5% MgCl_2_ (258 mM)	266.35	36.67	7.26
5% MgCl_2_ (521 mM)	160.44	30.86	5.20
10% MgCl_2_ (1052 mM)	68.84	28.59	2.41
1.9% Ca(ClO_4_)_2_ (103 mM)	282.95	46.84	6.04
3.8% Ca(ClO_4_)_2_ (207 mM)	113.71	17.93	6.34
7.7% Ca(ClO_4_)_2_ (418 mM)	18.33	15.92	1.15
2.5% Mg(ClO_4_)_2_ (110 mM)	174.37	33.35	5.23
5% Mg(ClO_4_)_2_ (222 mM)	51.86	14.11	3.68
10% Mg (ClO_4_)_2_ (448 mM)	21.8	16.94	1.29

### Survival assessment with CFU counts

Assessment of bacterial survival was performed with CFU counts on Day 0, 5, 10, and 30 (Fig. 2). Independent of the temperature or the treatment, the number of colonies declined over time and was lower at Day 30 compared to Day 5 (t-test with p-value = 0.0029, see Supplementary File 1, Sect. “Results”).

In most of the cases, with the exception of the cultures in control conditions and those incubated with 5% w/v (222 mM) Mg(ClO_4_)_2_, the CFU counts revealed higher survival at 0 °C compared to 25 °C. At 0 °C (Fig. 2D), cells incubated without salt amendment showed a steep reduction in CFU number between Day 0 and Day 5, but the number of counted colonies remained relatively stable in the CFU counts at the 10th and 30th day of the experiment. Cells incubated with MgCl_2_ and Ca(ClO_4_)_2_ showed better survival even at Day 30, while for those incubated in media containing Mg(ClO_4_)_2_ colonies were only observed in the 2.5% w/v (110 mM) concentration treatment. With CaCl_2_, survival at Day 30 was only observed at 1.8% w/v (165 mM).

Cultures incubated at 25 °C (Fig. 2B) revealed a different pattern. First, the CFU number of the unamended culture remained stable throughout the experiment, but the number was lower for all amended cultures. The CFU number declined further with higher concentration and over time, and no CFUs were observed at Day 30 for cultures amended with perchlorate. The cultures amended with perchlorate showed lower CFU number compared to cultures amended with similar concentration of chloride.

The CFU counts for both temperature treatments were compared with the average CO_2_ production during the 30 days of the experiment (Figs. 2A and C). The results seem to correlate as in the cases where CFUs were observed until the last experimental day the CO_2_ concentration curves seem to also follow an increasing trend. In the case where colonies were not present, the CO_2_ production rate also seems to be lower and in most cases the CO_2_ concentration curves quickly reached a plateau.

## Discussion

The main objective of this study was to assess whether the bacterium *Deinococcus radiodurans* can remain metabolically functional under the combined effect of low temperature and presence of chloride and perchlorate salts. Previous studies have shown survival of microorganisms in perchlorate concentrations higher than 10% w/v (Heinz et al. 2020), but little is known about the effect of high perchlorate concentration on the metabolic activity of microorganisms.

We found that *D. radiodurans* has reduced metabolic activity when exposed to a combination of high salt concentration (calcium or magnesium chloride or perchlorate) and low incubation temperature. The different response to chloride and perchlorate salts indicates that the negative effect of perchlorate on *D. radiodurans* activity is due to both a general effect of enhanced osmolarity and a specific effect of perchlorates.

The average CO_2_ production rate was more than ten times larger for the control sample incubated at 25 °C compared to the one at 0 °C, but the ratio for activity at the two temperatures (Table 2) decreased with increasing salt concentration revealing an interactive effect of salt concentration on the temperature response of *D. radiodurans* activity. Such effects have already been observed in previous experiments where microorganisms performed better in medium range temperatures and less saline environments than in a combination of cold and highly saline environment (Diaz and Schulze-Makuch 2006; Harris et al. 2021). The lower limit for *D. radiodurans’* activity has not yet been determined, but the optimal growth temperature has been suggested to be 37 °C (Holland et al. 2006). Nevertheless, viability of Deinococci was observed at temperatures down to −35 °C as mentioned above (Diaz and Schulze-Makuch 2006) and we show activity at 0 °C.

While metabolic activity was several-fold higher at 25 °C compared to 0 °C in most cases, varying from 12 times larger for the control treatment to an almost equal rate in the higher perchlorate and CaCl_2_ treated ones (Table 2), we generally observed longer survival and higher CFU numbers from cultures incubated at 0 °C than those growing at 25 °C (Fig. 2). This was mainly observed for cultures treated with both chlorides and Ca(ClO_4_)_2_. For Mg(ClO_4_)_2,_ the results were slightly different as longer survival was seen for the 25 °C treated cultures, albeit at lower colony numbers. This might stem from competition of *Deinococci* at 25 °C that is favorable for their growth (Cox and Battista 2005) and may lead to depletion of essential nutrients.

In the case of cultures incubated at 0 °C, we observed some increase in the CO_2_ concentration between the 10th and 20th day (Fig. 2C) even in the cases where no survival was observed in the CFU counts (Fig. 2D). We assume that this might be due to release of gas from the liquid phase as the cultures were not shaked and thus gas might have been trapped in the liquid and not released earlier. This might be a result of higher solubility of CO_2_ in the liquid phase at low temperature. This pattern was not observed in such a prominent way in the cultures incubated at 25 °C (Figs. 2A and B), probably due to the higher temperature that does not facilitate gas retention in such extent (Adnan et al. 2020).

Previous works have tested the survival or growth of other microorganisms amended with perchlorate salts at different temperatures with different results. The methanogenic archaeon *Methanosarcina barkeri* was cultured with 20 mM of calcium, magnesium or 10 mM of sodium perchlorate at 30 and 0 °C respectively, with methane production in all cases being reduced when incubating with perchlorates and a greater effect was observed, similarly to our work, for the lower incubation temperature (Harris et al. 2021). Perchlorate salts were also used (calcium, magnesium or sodium) in different concentrations, 0.6 wt% for all the three salts and up to 1 wt% for magnesium perchlorate, and reduced the viability of *Bacillus subtillis* in all cases (Wadsworth and Cockell 2017). In the same work, a change of incubation temperature from 25 to 4 °C in one of the treatments indicated that lower temperature might be able to decrease *B. subtilis* mortality rates in certain cases as we observed for *D. radiodurans*.

In the case of photosynthetic bacteria, the cyanobacterium *Chroococcidopsis sp*. was able to grow in up to 50 mΜ of magnesium and calcium perchlorate and at 100 mΜ οf sodium perchlorate (Billi et al. 2021), while another study tested 17 different cyanobacterial species with magnesium perchlorate at concentrations varying between 0 and 1% w/v at 21 °C and found five species that were growing at the highest salt concentration (Rzymski et al. 2022). Similar data have also been obtained from the study of different archaea (Serrano et al. 2019). This study suggestsed that the process of methanogenesis in those microorganisms is reduced or fully halted when exposed to concentrations of magnesium perchlorate up to 500 mM.

We observed in most cases that chloride-incubated cultures showed longer viability than those incubated with perchlorates indicating a lower tolerance of *D.radiodurans* to the perchlorate anion (ClO_4_^−^) compared to chloride (Cl^−^). Similar observations was done on the growth of the bacterium *Planococcus halocryophilus* (Heinz et al. 2019) at 25° and 4 °C. The concentrations used varied as the maximum tolerated concentration was looked for. For the chloride-containing salts this concentration varied between 0.78 – 2.79 mol L^−1^ (NaCl and CaCl_2_ respectively) while for the perchlorate ones it was much lower (maximum 1.11 mol L^−1^ for NaClO_4_ and minimum 0.13 mol L^−1^ for Ca(ClO_4_)_2_). This increase to chloride-containing salts tolerance was observed after inoculation of *P. halocryophilus* with gradually increasing salt concentrations. The bacterium was less tolerant to calcium-containing salts although an increased tolerance was observed at 4 °C while for salts with sodium and magnesium, maximum tolerance was found at 25 °C. The ability of *D. radiodurans* to sustain activity and show survival up to 5%w/v perchlorate (222 mM Mg(ClO_4_)_2_) is remarkable and at the upper range observed for survival and growth of bacteria and archaea.

The growth rate of the halophile archaeon *Halorubrum lacusprofundi* was reduced by 50% with concentrations of 0.3 M of sodium or 0.1 M of calcium perchlorate compared to non-amended controls. In the same work when MgCl_2_ was used the growth rate remained relatively unaffected until 0.9 M concentration of MgCl_2_ and later a 40% reduction of the growth rate was observed at 1.2 M of the salt (Laye and DasSarma 2018). The above results are in agreement with our observation that perchlorate inhibits more the survival and growth of *D.radiodurans* compared to chloride. It is also an indication that the cation type and its size and charge might play an important role especially at high concentrations.

These studies do not consider the complete array of adversities that would apply on the Martian surface – unless modified by possible future human activity. The current conditions on the Martian surface, e.g., high radiation level, low temperature, low water availability and high concentrations of perchlorate salts, are hostile for known Earth organisms (Catling 2014; Cockell et al. 2000; Hassler et al. 2013; Kieffer 1976; Smith et al. 2004). In contrast to the incubation conditions in our and other studies, the Martian atmosphere lacks any significant concentration of atmospheric oxygen. However, perchlorate is a strong electron acceptor and reduction of perchlorate in combination with oxidation of an organic or inorganic electron donor may offer an alternative to reduction of oxygen on the Martian surface (Coates and Achenbach 2004). Several bacteria, mainly belonging to the Proteobacteria phylum, are known to carry out this process. Here, two enzymes play a central role in the process, first perchlorate dismutase reduces perchlorate to chlorite, which is further reduced to chlorine and O_2_ by the enzyme chlorite dismutase. No published reference has been found for the existence of chlorite or perchlorate dismutase analogues on *D.radiodurans.* Nevertheless, two entries on the existence of a chlorite dismutase gene can be found in the NCBI repository with Reference Sequences NZ_CP038663.1 and NZ_CP031500.1 (Clark et al. 2016).

In conclusion, perchlorate salts strongly affected *D. radiodurans* metabolic activity and survival partly due to osmotic stress and partly due to specific effects of perchlorate. However, low activity rates and survival were still observed at 5% w/v (222 mM) magnesium perchlorate. Low temperature lowered the activity, but enhanced survival of *D. radiodurans* indicating its ability to survive and sustain activity under extremities partly simulating Martian conditions.

## Supplementary Information

Below is the link to the electronic supplementary material.

Supplementary file1 (PDF 1853 KB)

Supplementary file2 (PDF 54 KB)

Supplementary file3 (PDF 51 KB)

## Data Availability

Data are available within the article and the supplementary material. Specific data are available in tabular form upon request.
