# Supplementary material for: Effects of temperature, chloride and perchlorate salt concentration on the metabolic activity of Deinococcus radiodurans
Source: Extremophiles. 2024 Jul 24;28(3):34. doi: 10.1007/s00792-024-01351-5 (PMC11266278; doi:10.1007/s00792-024-01351-5)
Supplement: Supplementary file 1 — Supplementary file1 (PDF 1853 KB) [file 792_2024_1351_MOESM1_ESM.pdf]

Supplementary Material

Section 1

Pre-experimental tests and preculture assessment

Table 1| Optical densities of cultures grown at 25°C with shaking at 40rpm for 10 days. There were two liquid precultures were grown in the above conditions. The optical density was measured twice for each culture.

Day	Culture	Optical Density (O.D.)	Average O.D.	O.D. Standard Deviation
1	1	0.4281	0.3923	0.0442
1	1	0.4325		
1	2	0.3485		
1	2	0.3599		
2	1	0.8059	0.7727	0.0273
2	1	0.7925		
2	2	0.7523		
2	2	0.7399		
3	1	1.1122	1.0928	0.0126
3	1	1.0864		
3	2	1.0944		
3	2	1.0783		
4	1	1.3479	1.3701	0.0419
4	1	1.4059		
4	2	1.414		
4	2	1.3124		
5	1	1.5922	1.6421	0.0484
5	1	1.5952		
5	2	1.69		
5	2	1.6911		
6	1	1.8119	1.8003	0.0137
6	1	1.7948		
6	2	1.8141		
6	2	1.7805		
8	1	2.0367	2.0761	0.0279
8	1	2.0981		
8	2	2.0934		
8	2	2.3639		
10	1	2.2137	2.2804	0.0608
10	1	2.2299		
10	2	2.318		
10	2	2.3601		

Table 2| CFU counts of cultures grown at 25°C with shaking at 40rpm for 10 days. There were two liquid precultures were grown in the above conditions. The dilutions were made starting with 10µl of initial culture diluted in total 100µl.

Day	Culture	Dilution	Number of Counted Colonies	CFU x 10^4/ ml	Average CFU X 10^4 / ml	Standard Deviation CFU X 10^4 / ml
1	1	10^{-1}	780	78	153.07	57.58
1	1	10^{-2}	170	170		
1	1	10^{-2}	219	219		
1	2	10^{-1}	744	74,4		
1	2	10^{-2}	167	167		
1	2	10^{-2}	210	210		
2	1	10^{-1}	1444	144.4	244.17	82.08
2	1	10^{-2}	215	215		
2	1	10^{-2}	271	271		
2	2	10^{-1}	1596	159,6		
2	2	10^{-2}	384	384		
2	2	10^{-2}	291	291		
3	1	10^{-2}	672	672	540.83	173.16
3	1	10^{-2}	492	492		
3	1	10^{-3}	85	850		
3	2	10^{-2}	317	317		
3	2	10^{-2}	374	474		
3	2	10^{-3}	44	440		
4	1	10^{-2}	231	231	407.17	195.91
4	1	10^{-2}	222	222		
4	1	10^{-3}	73	730		
4	2	10^{-2}	221	221		
4	2	10^{-2}	499	499		
4	2	10^{-3}	54	540		
5	1	10^{-1}	2136	213.6	549.09	272.81
5	1	10^{-2}	315	315		
5	1	10^{-2}	464	464		
5	1	10^{-3}	68	680		
5	2	10^{-2}	614	614		
5	2	10^{-2}	447	447		
5	2	10^{-3}	111	1110		
6	1	10^{-2}	586	586	1196	418.97
6	1	10^{-3}	165	1650		
6	1	10^{-3}	167	1670		
6	2	10^{-2}	900	900		
6	2	10^{-3}	90	900		
6	2	10^{-3}	147	1470		
8	1	10^{-2}	505	505	1714.5	909.17
8	1	10^{-3}	266	2660		
8	1	10^{-3}	202	2020		
8	2	10^{-2}	682	682		
8	2	10^{-3}	290	2900		
8	2	10^{-3}	152	1520		
10	1	10^{-2}	959	959	2027.83	901.66
10	1	10^{-3}	295	2950		
10	1	10^{-3}	237	2370		
10	2	10^{-2}	838	838		
10	2	10^{-3}	318	3180		
10	2	10^{-3}	187	1870		

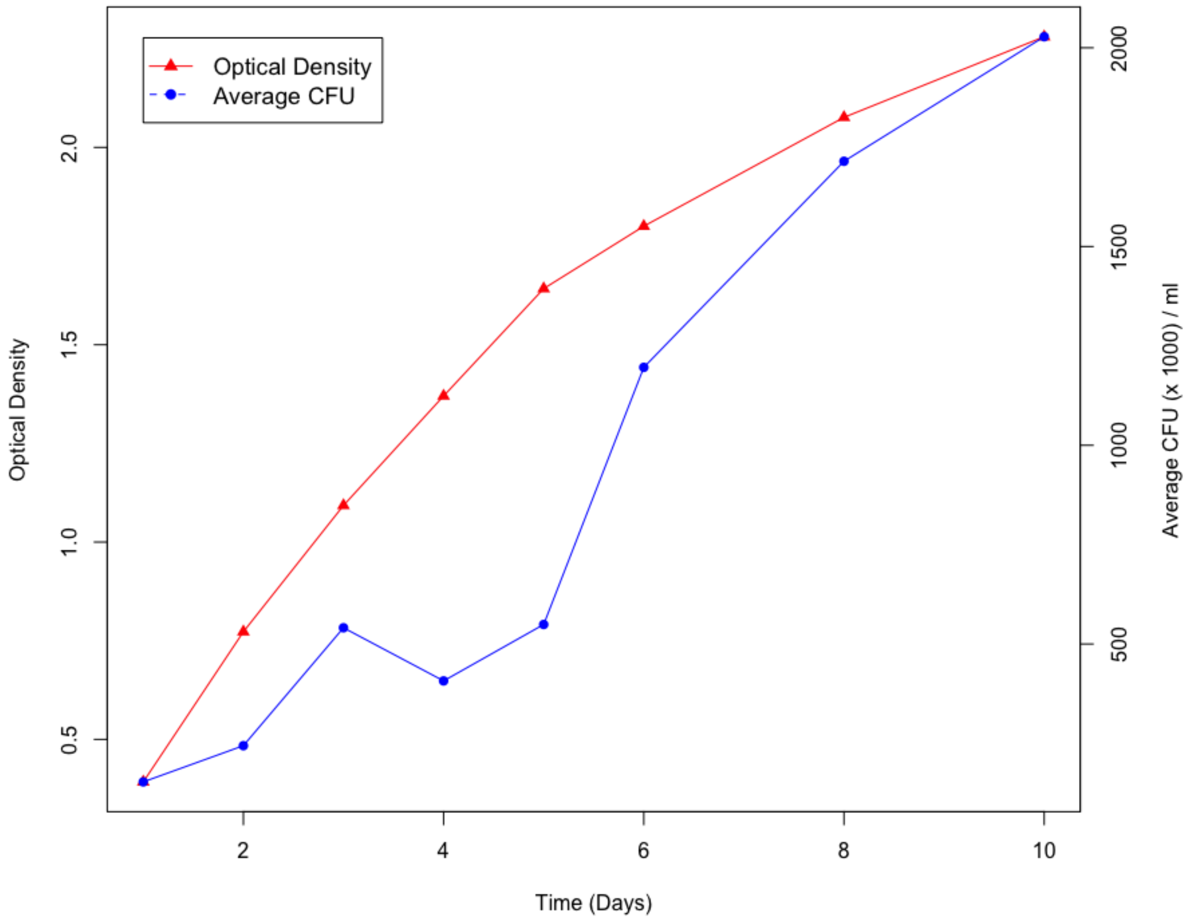

Figure 1| Growth assessment of Deinococcus radiodurans cultures grown at 25°C with shaking at 40rpm for 10 days. Average optical density is represented by the red line with red triangles. Average CFU count is represented by the blue line with blue dots.

Table 3| Optical density of liquid precultures used for the experiments. The precultures used for the 0°C experiments were grown for a total of 10 days. The precultures used for the 25°C experiments were grown for a total of 6 days.

Liquid Preculture number	Temperature to be incubated (°C)	Optical Density
1	0	2.683
1	0	2.6054
2	0	2.3095
2	0	2.3585
1	25	1.9032
1	25	1.8612
2	25	1.7464
2	25	1.7202

The precultures were not mixed together. 1ml of the first preculture was added in the vials. As the amount of preculture 1 was not enough for all the culture vials in both temperatures, preculture 2 was added in the rest of the vials. Both precultures 2 were added only in vials used for the CFU counts.

We calculated the amount of salt needed in each jar containing liquid culture medium and liquid culture in final volume of 10ml. The salt was added along with the liquid medium in the jars (volume of 9 ml in total) before the addition of liquid culture (1ml in total).

For a final salt concentration of:

- 2.5% w/v, we added 0.245 g of salt.
- 5% w/v, we added 0.495 g of salt.
- 10% w/v, we added 0.999 g of salt.

Based on the knowledge of each salts' molecular weight we calculated the amount of each salt per treatment. The results are given in **Table 4** below.

Table 4| Conversion of salt concentrations (%w/v) to amount of salt (mmol).

Concentration (%w/v)	Salt	Amount of Substance (mmol)
1.8	$CaCl_2$	1.65
2.5	$MgCl_2$	2.58
1.9	$Ca(ClO_4)_2$	0.78
2.5	$Mg(ClO_4)_2$	1.10
3.7	$CaCl_2$	3.37
5	$MgCl_2$	5.21
3.8	$Ca(ClO_4)_2$	1.59
5	$Mg(ClO_4)_2$	2.22
7.5	$CaCl_2$	6.80
10	$MgCl_2$	10.52
7.7	$Ca(ClO_4)_2$	3.21
10	$Mg(ClO_4)_2$	4.48

Section 2

CFU Counts

Table 5| Dilution percentage, colony count on plates and CFU assessment and statistical results for Control cultures at 25°C.

Day of Incubation[1]	Dilution	Number of Counted Colonies	Number of Cells per ml (x 10^4)	Average	Standard Deviation	Standard Error
0 (1)	10^{-2}	925	925	1492.93	1012.25	72.30
0 (1)	10^{-2}	952	952			
0 (1)	10^{-3}	171	1710			
0 (1)	10^{-3}	179	1790			
0 (1)	10^{-3}	197	1970			
0 (1)	10^{-4}	11	1100			
0 (1)	10^{-4}	13	1300			
0 (1)	10^{-4}	16	1600			
0 (1)	10^{-4}	47	4700			
0 (2)	10^{-2}	864	864			
0 (2)	10^{-2}	580	580			
0 (2)	10^{-3}	75	750			
0 (2)	10^{-3}	123	1230			
0 (2)	10^{-3}	143	1430			
5	10^{-2}	1144	1144	2113	662.4	270.42
5	10^{-2}	1904	1904			
5	10^{-3}	252	2520			
5	10^{-3}	241	2410			
5	10^{-4}	17	1700			
5	10^{-4}	30	3000			
10	10^{-2}	270	270	1557.5	886.81	443.40
10	10^{-3}	180	1800			
10	10^{-3}	186	1860			
10	10^{-4}	23	2300			
10	10^{-4}	0	0 [2]			
30	10^{-2}	910	910	1263.33	552.11	225.40
30	10^{-2}	1070	1070			
30	10^{-3}	106	1060			
30	10^{-3}	144	1440			
30	10^{-4}	8	800			
30	10^{-4}	23	2300			

[1] As two different initial precultures were added in the experimental vials, both samples were taken from each on and are presented with the numbers 1 and 2 respectively
[2] In the case that one single count out of the total counts for a treatment was 0, was excluded from the statistical testing.

Table 6| Dilution percentage, colony count on plates and CFU assessment and statistical results for Control cultures at 0°C.

Day of Incubation[1]	Dilution	Number of Counted Colonies	Number of Cells per ml ($x 10^4$)	Average	Standard Deviation	Standard Error
0 (1)	10^{-2}	1252	1252	3052.44	1075.02	368.76
0 (1)	10^{-2}	966	966			
0 (1)	10^{-3}	272	2720			
0 (1)	10^{-3}	517	5170			
0 (1)	10^{-3}	388	3880			
0 (1)	10^{-4}	56	5600			
0 (1)	10^{-4}	40	4000			
0 (1)	10^{-4}	42	4200			
0 (1)	10^{-2}	1600	1600			
0 (2)	10^{-2}	872	872			
0 (2)	10^{-3}	280	2899			
0 (2)	10^{-3}	308	3080			
0 (2)	10^{-3}	270	2700			
0 (2)	10^{-4}	40	4000			
0 (2)	10^{-4}	17	1700			
0 (2)	10^{-4}	42	4200			
5	10^{-2}	278	278	421.83	126.5	51.64
5	10^{-2}	383	383			
5	10^{-3}	46	460			
5	10^{-3}	61	610			
5	10^{-4}	3	300			
5	10^{-4}	5	500			
10	10^{-2}	179	179	463.83	448.1	182.94
10	10^{-2}	174	174			
10	10^{-3}	41	410			
10	10^{-3}	12	120			
10	10^{-4}	13	1300			
10	10^{-4}	6	600			
30	10^{-2}	508	508	294.2	167.92	75.09
30	10^{-2}	183	183			
30	10^{-3}	26	260			
30	10^{-3}	42	420			
30	10^{-4}	1	100			
30	10^{-4}	0	0 [2]			

[1] As two different initial precultures were added in the experimental vials, both samples were taken from each on and are presented with the numbers 1 and 2 respectively

[2] In the case that one single count out of the total counts for a treatment was 0, was excluded from the statistical testing.

Table 7| Dilution percentage, colony count on plates and CFU assessment and statistical results for cultures treated with $CaCl_2$ at 25°C.

Salt concentration (% w/v)	Day of Incubation	Dilution	Number of Counted Colonies	Number of Cells per ml (x 10^4)	Average	Standard Deviation	Standard Error
1.8	5	10^{-1}	580	58	102.25	67.59	33.79
1.8	5	10^{-2}	56	56			
1.8	5	10^{-2}	95	95			
1.8	5	10^{-3}	0	0[1]			
1.8	5	10^{-3}	20	200			
3.7	5	10^{-1}	262	26.2	34.44	26.72	11.95
3.7	5	10^{-2}	24	24			
3.7	5	10^{-2}	32	32			
3.7	5	10^{-3}	8	80			
3.7	5	10^{-3}	1	10			
7.5	5	10^{-1}	158	15.8	10.40	5.37	2.69
7.5	5	10^{-1}	108	10.8			
7.5	5	10^{-2}	12	12			
7.5	5	10^{-2}	3	3			
1.8	10	10^{-1}	295	29.5	57.5	39.79	17.79
1.8	10	10^{-2}	85	85			
1.8	10	10^{-2}	13	13			
1.8	10	10^{-3}	5	50			
1.8	10	10^{-3}	11	110			
3.7	10	10^{-1}	33	3,3	10.33	5.11	2.56
3.7	10	10^{-2}	13	13			
3.7	10	10^{-2}	15	15			
3.7	10	10^{-3}	1	10			
3.7	10	10^{-3}	0	0[1]			
7.5	10	10^{-1}	11	1,1	2.33	1.43	0.83
7.5	10	10^{-1}	39	3,9			
7.5	10	10^{-2}	0	0[1]			
7.5	10	10^{-2}	2	2			
1.3	30	10^{-1}	276	27.6	29.92	9.82	4.39
1.8	30	10^{-2}	24	24			
1.8	30	10^{-2}	18	18			
1.8	30	10^{-3}	4	40			
1.8	30	10^{-3}	4	40			
3.7	30	10^{-1}	0	0	0	0	0
3.7	30	10^{-2}	0	0			
3.7	30	10^{-2}	0	0			
3.7	30	10^{-3}	0	0			
3.7	30	10^{-3}	0	0			
7.5	30	10^{-1}	0	0	0	0	0
7.5	30	10^{-1}	0	0			
7.5	30	10^{-2}	0	0			
7.5	30	10^{-2}	0	0			

[1] In the case that one single count out of the total counts for a treatment was 0, was excluded from the statistical testing.

Table 8| Dilution percentage, colony count on plates and CFU assessment and statistical results for cultures treated with $CaCl_2$ at 0°C.

Salt concentration (% w/v)	Day of Incubation	Dilution	Number of Counted Colonies	Number of Cells per ml (x 10^4)	Average	Standard Deviation	Standard Error
1.8	5	10^{-1}	650	65	235.67	166.42	67.94
1.8	5	10^{-2}	91	91			
1.8	5	10^{-2}	298	298			
1.8	5	10^{-3}	18	180			
1.8	5	10^{-3}	26	260			
1.8	5	10^{-3}	52	520			
3.7	5	10^{-1}	1481	148.1	213.53	47	23.50
3.7	5	10^{-2}	221	221			
3.7	5	10^{-2}	225	225			
3.7	5	10^{-3}	26	260			
7.5	5	10^{-1}	188	18.8	21.58	7.32	3.66
7.5	5	10^{-1}	205	20.5			
7.5	5	10^{-2}	32	32			
7.5	5	10^{-2}	15	15			
1.8	10	10^{-1}	1576	157.6	379.32	230.45	103.06
1.8	10	10^{-2}	236	236			
1.8	10	10^{-2}	253	253			
1.8	10	10^{-3}	68	680			
1.8	10	10^{-3}	57	570			
3.7	10	10^{-1}	416	41.6	56.52	42.14	18.85
3.7	10	10^{-2}	122	122			
3.7	10	10^{-2}	69	69			
3.7	10	10^{-3}	4	40			
3.7	10	10^{-3}	1	10			
7.5	10	10^{-1}	4	0.4	0.78	0.29	0.14
7.5	10	10^{-1}	7	0.7			
7.5	10	10^{-2}	1	1			
7.5	10	10^{-2}	1	1			
1.8	30	10^{-1}	448	44.8	71.56	22.65	10.13
1.8	30	10^{-2}	90	90			
1.8	30	10^{-2}	93	93			
1.8	30	10^{-3}	8	80			
1.8	30	10^{-3}	5	50			
3.7	30	10^{-1}	1	0.1	0.02	0.04	0.02
3.7	30	10^{-2}	0	0			
3.7	30	10^{-2}	0	0			
3.7	30	10^{-3}	0	0			
3.7	30	10^{-3}	0	0			
7.5	30	10^{-1}	0	0	0	0	0
7.5	30	10^{-1}	0	0			
7.5	30	10^{-2}	0	0			
7.5	30	10^{-2}	0	0			

Table 9| Dilution percentage, colony count on plates and CFU assessment and statistical results for cultures treated with $MgCl_2$ at 25°C.

Salt concentration (% w/v)	Day of Incubation	Dilution	Number of Counted Colonies	Number of Cells per ml (x 10^4)	Average	Standard Deviation	Standard Error
2.5	5	10^{-1}	412	41.2	64.44	27.02	12.08
2.5	5	10^{-2}	51	51			
2.5	5	10^{-2}	109	109			
2.5	5	10^{-3}	5	50			
2.5	5	10^{-3}	7	70			
5	5	10^{-1}	307	30.7	41.74	31.33	14.01
5	5	10^{-2}	13	13			
5	5	10^{-2}	55	55			
5	5	10^{-3}	9	90			
5	5	10^{-3}	2	20			
10	5	10^{-1}	235	23.5	33.98	14.17	7.09
10	5	10^{-1}	244	24.4			
10	5	10^{-2}	34	34			
10	5	10^{-2}	54	54			
2.5	10	10^{-1}	438	43.8	94.76	34.54	15.45
2.5	10	10^{-2}	95	95			
2.5	10	10^{-2}	105	105			
2.5	10	10^{-3}	9	90			
2.5	10	10^{-3}	14	140			
5	10	10^{-1}	205	20.5	38.50	15.79	9.12
5	10	10^{-2}	45	45			
5	10	10^{-2}	0	0[1]			
5	10	10^{-3}	5	50			
10	10	10^{-1}	11	1.1	0.53	0.61	0.30
10	10	10^{-1}	0	0			
10	10	10^{-2}	1	1			
10	10	10^{-2}	0	0[1]			
2.5	30	10^{-1}	72	7.2	17.55	9.47	4.73
2.5	30	10^{-2}	15	15			
2.5	30	10^{-2}	18	18			
2.5	30	10^{-3}	0	0			
2.5	30	10^{-3}	3	30			
5	30	10^{-1}	67	6.7	15.68	6.11	3.06
5	30	10^{-2}	19	19			
5	30	10^{-2}	17	17			
5	30	10^{-3}	2	20			
5	30	10^{-3}	0	0[1]			
10	30	10^{-1}	0	0	0	0	0
10	30	10^{-1}	0	0			
10	30	10^{-2}	0	0			
10	30	10^{-2}	0	0			

[1] In the case that one single count out of the total counts for a treatment was 0, was excluded from the statistical testing.

Table 10| Dilution percentage, colony count on plates and CFU assessment and statistical results for cultures treated with $MgCl_2$ at 0°C.

Salt concentration (% w/v)	Day of Incubation	Dilution	Number of Counted Colonies	Number of Cells per ml (x 10^4)	Average	Standard Deviation	Standard Error
2.5	5	10^{-1}	2768	276.8	235.76	158.96	71.09
2.5	5	10^{-2}	396	396			
2.5	5	10^{-2}	266	366			
2.5	5	10^{-3}	4	40			
2.5	5	10^{-3}	10	100			
5	5	10^{-1}	1796	179.6	285.32	117.82	52.69
5	5	10^{-2}	305	305			
5	5	10^{-2}	172	372			
5	5	10^{-3}	15	150			
5	5	10^{-3}	42	420			
10	5	10^{-1}	3060	306	365.45	100.13	50.07
10	5	10^{-1}	2568	256.8			
10	5	10^{-2}	432	432			
10	5	10^{-2}	467	467			
2.5	10	10^{-1}	1160	116	218	152.67	68.28
2.5	10	10^{-2}	280	280			
2.5	10	10^{-2}	64	64			
2.5	10	10^{-3}	18	180			
2.5	10	10^{-3}	45	450			
5	10	10^{-1}	468	46.8	233.96	154.93	69.29
5	10	10^{-2}	255	265			
5	10	10^{-2}	128	128			
5	10	10^{-3}	45	450			
5	10	10^{-3}	28	280			
10	10	10^{-1}	2180	218	311.20	156.38	78.19
10	10	10^{-1}	1528	152.8			
10	10	10^{-2}	374	374			
10	10	10^{-2}	500	500			
2.5	30	10^{-1}	1796	179.6	218.32	55.23	24.70
2.5	30	10^{-2}	192	192			
2.5	30	10^{-2}	230	230			
2.5	30	10^{-3}	18	180			
2.5	30	10^{-3}	31	310			
5	30	10^{-1}	1268	126.8	171.96	87.38	39.08
5	30	10^{-2}	99	99			
5	30	10^{-2}	104	104			
5	30	10^{-3}	29	290			
5	30	10^{-3}	24	240			
10	30	10^{-1}	1030	103	115.20	57.32	28.66
10	30	10^{-1}	1328	132.8			
10	30	10^{-2}	181	181			
10	30	10^{-2}	44	44			

Table 11| Dilution percentage, colony count on plates, CFU assessment and statistical results for cultures treated with $Ca(ClO_4)_2$ at 25°C.

Salt concentration (% w/v)	Day of Incubation	Dilution	Number of Counted Colonies	Number of Cells per ml (x 10^4)	Average	Standard Deviation	Standard Error
1.9	5	10^{-1}	536	53.6	46.65	6.66	3.33
1.9	5	10^{-2}	42	42			
1.9	5	10^{-2}	51	51			
1.9	5	10^{-3}	4	40			
1.9	5	10^{-3}	0	0[1]			
3.8	5	10^{-1}	368	36.8	49.16	20.10	8.99
3.8	5	10^{-2}	41	41			
3.8	5	10^{-2}	58	58			
3.8	5	10^{-3}	3	30			
3.8	5	10^{-3}	8	80			
7.7	5	10^{-1}	0	0	0	0	0
7.7	5	10^{-1}	0	0			
7.7	5	10^{-2}	0	0			
7.7	5	10^{-2}	0	0			
1.9	10	10^{-1}	0	0	0	0	0
1.9	10	10^{-2}	0	0			
1.9	10	10^{-2}	0	0			
1.9	10	10^{-3}	0	0			
1.9	10	10^{-3}	0	0			
3.8	10	10^{-1}	15	1.5	0.7	0.97	0.44
3.8	10	10^{-2}	2	2			
3.8	10	10^{-2}	0	0			
3.8	10	10^{-3}	0	0			
3.8	10	10^{-3}	00	0			
7.7	10	10^{-1}	0	0	0	0	0
7.7	10	10^{-1}	0	0			
7.7	10	10^{-2}	0	0			
7.7	10	10^{-2}	0	0			
1.9	30	10^{-1}	0	0	0.25	0.50	0.25
1.9	30	10^{-2}	0	0			
1.9	30	10^{-2}	1	1			
1.9	30	10^{-3}	0	0			
3.8	30	10^{-1}	0	0	0	0	0
3.8	30	10^{-2}	0	0			
3.8	30	10^{-2}	0	0			
3.8	30	10^{-3}	0	0			
3.8	30	10^{-3}	0	0			
7.7	30	10^{-1}	0	0	0	0	0
7.7	30	10^{-1}	0	0			
7.7	30	10^{-2}	0	0			
7.7	30	10^{-2}	0	0			

[1] In the case that one single count out of the total counts for a treatment was 0, was excluded from the statistical testing.

Table 12| Dilution percentage, colony count on plates, CFU assessment and statistical results for cultures treated with $Ca(ClO_4)_2$ at 0°C.

Salt concentration (% w/v)	Day of Incubation	Dilution	Number of Counted Colonies	Number of Cells per ml (x 10^4)	Average	Standard Deviation	Standard Error
1.9	5	10^{-1}	2568	256.8	444.70	134.13	67.07
1.9	5	10^{-2}	518	518			
1.9	5	10^{-2}	444	444			
1.9	5	10^{-3}	56	560			
3.8	5	10^{-1}	2448	244.8	313.16	66.96	29.95
3.8	5	10^{-2}	273	273			
3.8	5	10^{-2}	368	368			
3.8	5	10^{-3}	40	400			
3.8	5	10^{-3}	28	280			
7.7	5	10^{-1}	1	0.1	0.1	0.14	0.07
7.7	5	10^{-1}	3	0.3			
7.7	5	10^{-2}	0	0			
7.7	5	10^{-2}	0	0			
1.9	10	10^{-1}	448	44.8	105.36	75.48	33.75
1.9	10	10^{-2}	20	20			
1.9	10	10^{-2}	122	122			
1.9	10	10^{-3}	21	210			
1.9	10	10^{-3}	13	130			
3.8	10	10^{-1}	1076	107.6	59.12	41.49	18.56
3.8	10	10^{-2}	40	40			
3.8	10	10^{-2}	28	28			
3.8	10	10^{-3}	10	100			
3.8	10	10^{-3}	4	40			
7.7	10	10^{-1}	1	0.1	0.025	0.05	0.025
7.7	10	10^{-1}	0	0			
7.7	10	10^{-2}	0	0			
7.7	10	10^{-2}	0	0			
1.9	30	10^{-1}	113	11.3	12.83	8.12	2.06
1.9	30	10^{-2}	18	18			
1.9	30	10^{-2}	2	2			
1.9	30	10^{-3}	0	0 [1]			
1.9	30	10^{-3}	2	20			
3.8	30	10^{-1}	90	9	13	5.05	2.26
3.8	30	10^{-2}	15	15			
3.8	30	10^{-2}	21	21			
3.8	30	10^{-3}	1	10			
3.8	30	10^{-3}	1	10			
7.7	30	10^{-1}	0	0	0	0	0
7.7	30	10^{-1}	0	0			
7.7	30	10^{-2}	0	0			
7.7	30	10^{-2}	0	0			

[1] In the case that one single count out of the total counts for a treatment was 0, was excluded from the statistical testing.

Table 13| Dilution percentage, colony count on plates, CFU assessment and statistical results for cultures treated with $Mg(ClO_4)_2$ at 25°C.

Salt concentration (% w/v)	Day of Incubation	Dilution	Number of Counted Colonies	Number of Cells per ml (x 10^4)	Average	Standard Deviation	Standard Error
2.5	5	10^{-1}	40	4	16	10.80	5.40
2.5	5	10^{-2}	13	13			
2.5	5	10^{-2}	17	17			
2.5	5	10^{-3}	3	30			
2.5	5	10^{-3}	0	0			
5	5	10^{-1}	201	20.1	29.42	12.78	5.72
5	5	10^{-2}	51	51			
5	5	10^{-2}	26	26			
5	5	10^{-3}	2	20			
5	5	10^{-3}	3	30			
10	5	10^{-1}	8	0.8	0.20	0.40	0.2
10	5	10^{-1}	0	0			
10	5	10^{-2}	0	0			
10	5	10^{-2}	0	0			
2.5	10	10^{-1}	35	3.5	2.63	2.98	1.49
2.5	10	10^{-2}	4	4			
2.5	10	10^{-2}	5	5			
2.5	10	10^{-3}	0	0			
2.5	10	10^{-3}	1	10			
5	10	10^{-1}	5	0.5	3.13	4.59	2.29
5	10	10^{-2}	1	1			
5	10	10^{-2}	1	1			
5	10	10^{-3}	1	10			
10	10	10^{-1}	0	0	0	0	0
10	10	10^{-1}	0	0			
10	10	10^{-2}	0	0			
10	10	10^{-2}	0	0			
2.5	30	10^{-1}	6	0.6	0.12	0.27	0.12
2.5	30	10^{-2}	0	0			
2.5	30	10^{-2}	0	0			
2.5	30	10^{-3}	0	0			
2.5	30	10^{-3}	0	0			
5	30	10^{-1}	0	0	0	0	0
5	30	10^{-2}	0	0			
5	30	10^{-2}	0	0			
5	30	10^{-3}	0	0			
5	30	10^{-3}	0	0			
10	30	10^{-1}	0	0	0	0	0
10	30	10^{-1}	0	0			
10	30	10^{-2}	0	0			
10	30	10^{-2}	0	0			

Table 14| Dilution percentage, colony count on plates, CFU assessment and statistical results for cultures treated with $Mg(ClO_4)_2$ at 0°C.

Salt concentration (% w/v)	Day of Incubation	Dilution	Number of Counted Colonies	Number of Cells per ml (x 10^4)	Average	Standard Deviation	Standard Error
2.5	5	10^{-1}	2864	286.4	299.48	153.17	68.50
2.5	5	10^{-2}	461	461			
2.5	5	10^{-2}	550	550			
2.5	5	10^{-3}	71	710			
2.5	5	10^{-3}	49	490			
5	5	10^{-1}	7	0,7	0.14	0.31	0.14
5	5	10^{-2}	0	0			
5	5	10^{-2}	0	0			
5	5	10^{-3}	0	0			
5	5	10^{-3}	0	0			
10	5	10^{-1}	0	0	0	0	0
10	5	10^{-1}	0	0			
10	5	10^{-2}	0	0			
10	5	10^{-2}	0	0			
2.5	10	10^{-1}	1492	149.2	163.04	63.75	28.51
2.5	10	10^{-2}	178	178			
2.5	10	10^{-2}	218	218			
2.5	10	10^{-3}	6	60			
2.5	10	10^{-3}	21	210			
5	10	10^{-1}	0	0	0	0	0
5	10	10^{-2}	0	0			
5	10	10^{-2}	0	0			
5	10	10^{-3}	0	0			
5	10	10^{-3}	0	0			
10	10	10^{-1}	0	0	0	0	0
10	10	10^{-1}	0	0			
10	10	10^{-2}	0	0			
10	10	10^{-2}	0	0			
2.5	30	10^{-1}	0	0	0.2	0.45	0.20
2.5	30	10^{-2}	1	1			
2.5	30	10^{-2}	0	0			
2.5	30	10^{-3}	0	0			
2.5	30	10^{-3}	0	0			
5	30	10^{-1}	0	0	0	0	0
5	30	10^{-2}	0	0			
5	30	10^{-2}	0	0			
5	30	10^{-3}	0	0			
5	30	10^{-3}	0	0			
10	30	10^{-1}	0	0	0	0	0
10	30	10^{-1}	0	0			
10	30	10^{-2}	0	0			
10	30	10^{-2}	0	0			

Section 3

Effects of concentration and molar concentration on CO_2 production rate quotient

To statistically test the significance of salt concentration (% w/v) and salt molar concentration we performed one and two-way ANOVA statistical tests in R. The resulting p-value for concentration was **0.0003** while for the molar concentration it was **0.0241** which is still under the 0.05 significance threshold. When the two values were tested together in a two-way ANOVA, the p-value for concentration alone was found to be **0.000324** while for molar concentration it was 0.471 and for the combination of them was 0.102.

Student's test (t-test) on the average amount of colonies observed on the 5th and 30th day of the experiment.

To statistically test whether the average numbers of the colonies counted for all treatments in both temperatures differed between the 5th and the 30th day. For this reason, we performed a paired two sample t-test for means in **Excel**. The p-value acquired was 0.00294 which is below the significance threshold of 0.05. Thus, we assume that the means are not the same for the colonies (as the null hypothesis has been that no statistical difference between the two days' mean values exist).

Section 4

Concentrations of CO_2 per culture replicate

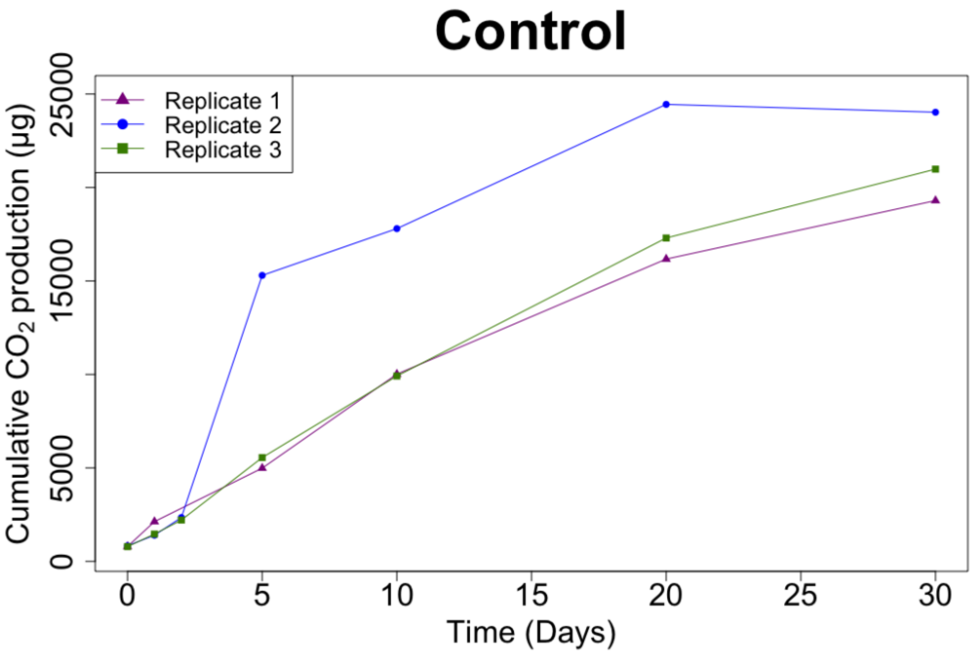

Figure 2| Cumulative CO_2 production lines for each replicate culture at 25°C with no salt in the growth medium (Control Cultures)

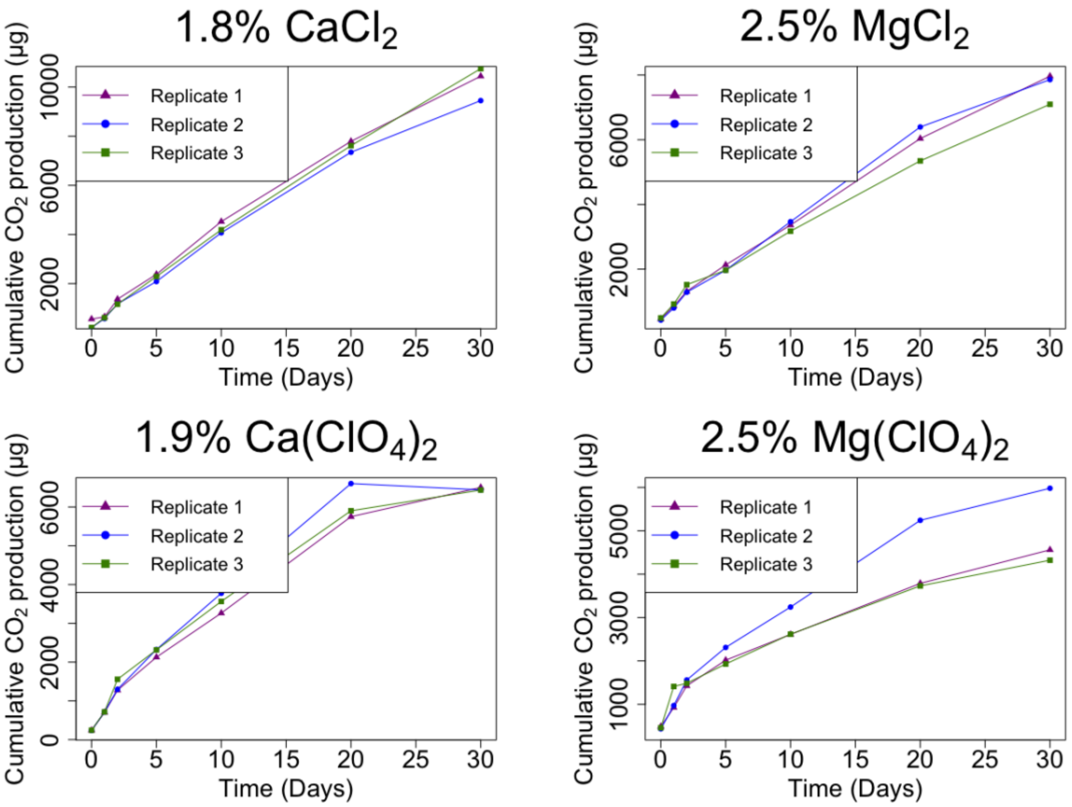

Figure 3| Cumulative CO_2 production lines for each replicate culture at 25°C with 2.5% concentration of each salt. The name of the salt type used in each case is on display in the main title on the top of each plot.

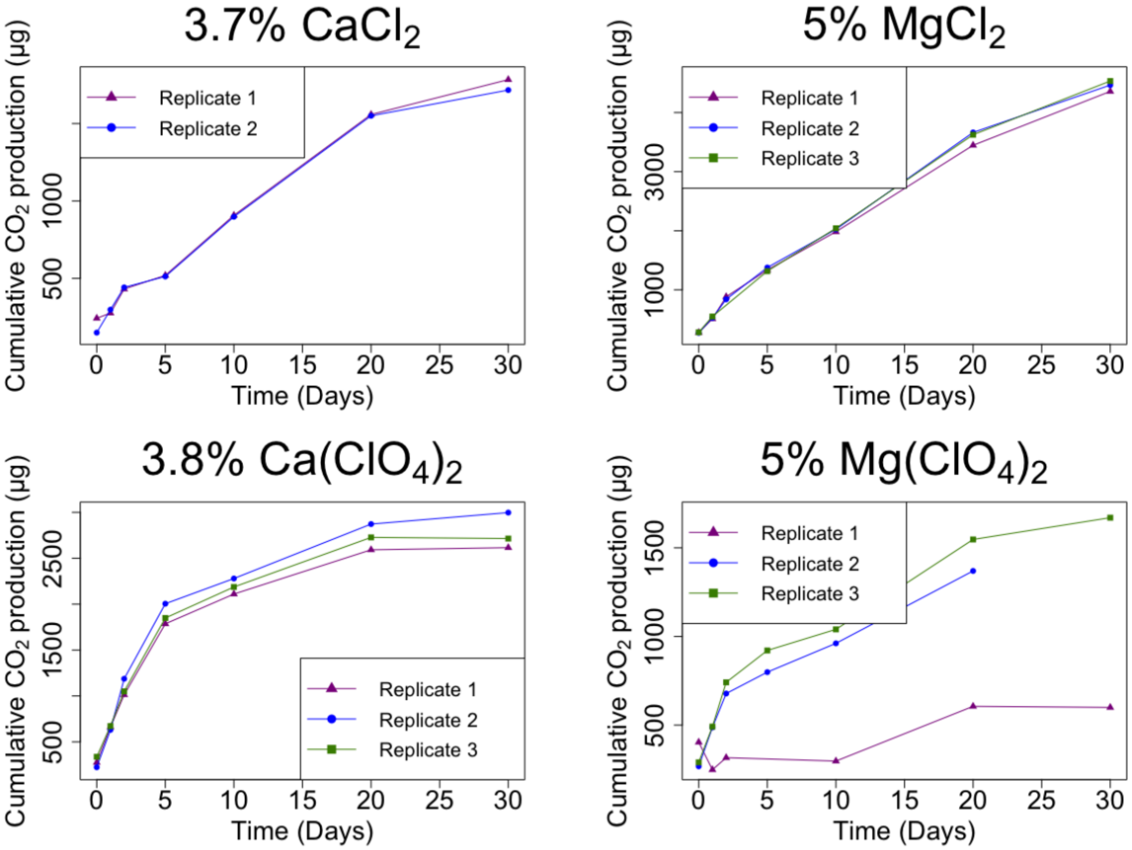

Figure 4| Cumulative CO_2 production lines for each replicate culture at 25°C with 5% concentration of each salt. The name of the salt type used in each case is on display in the main title on the top of each plot.

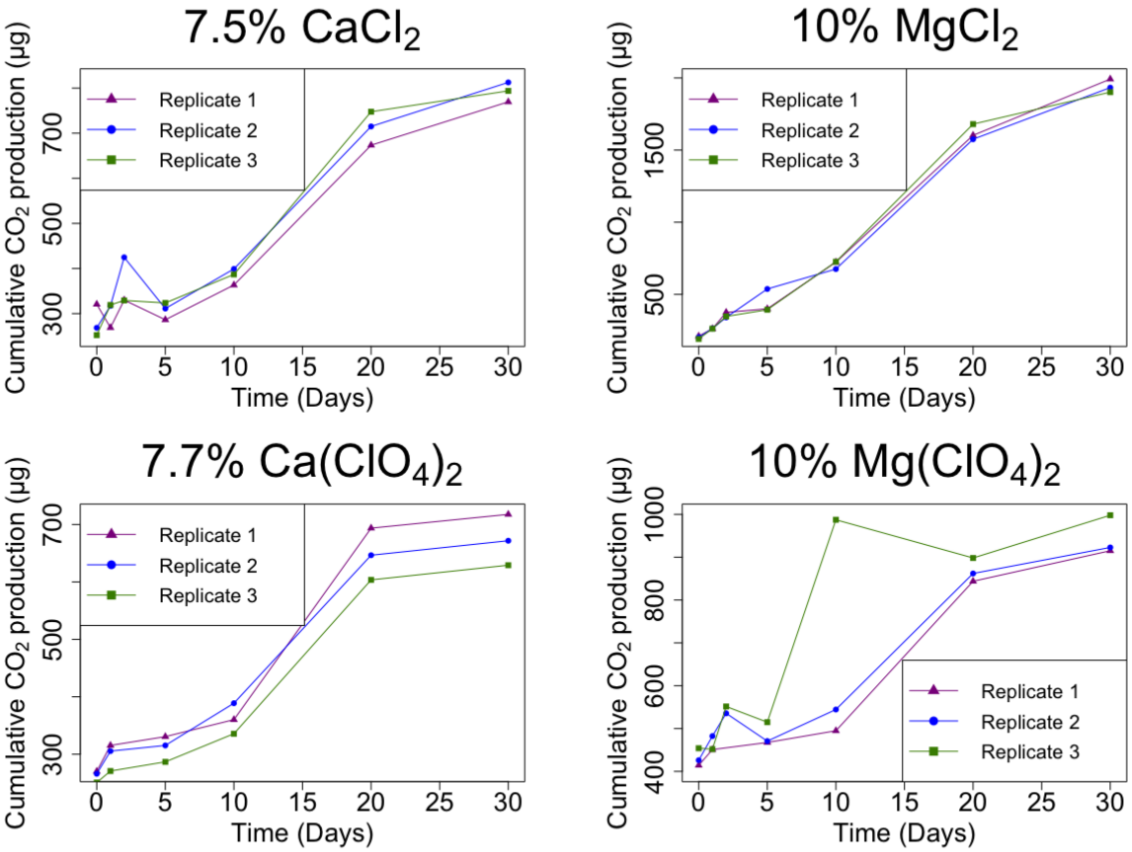

Figure 5| Cumulative CO_2 production lines for each replicate culture at 25°C with 10% concentration of each salt. The name of the salt type used in each case is on display in the main title on the top of each plot.

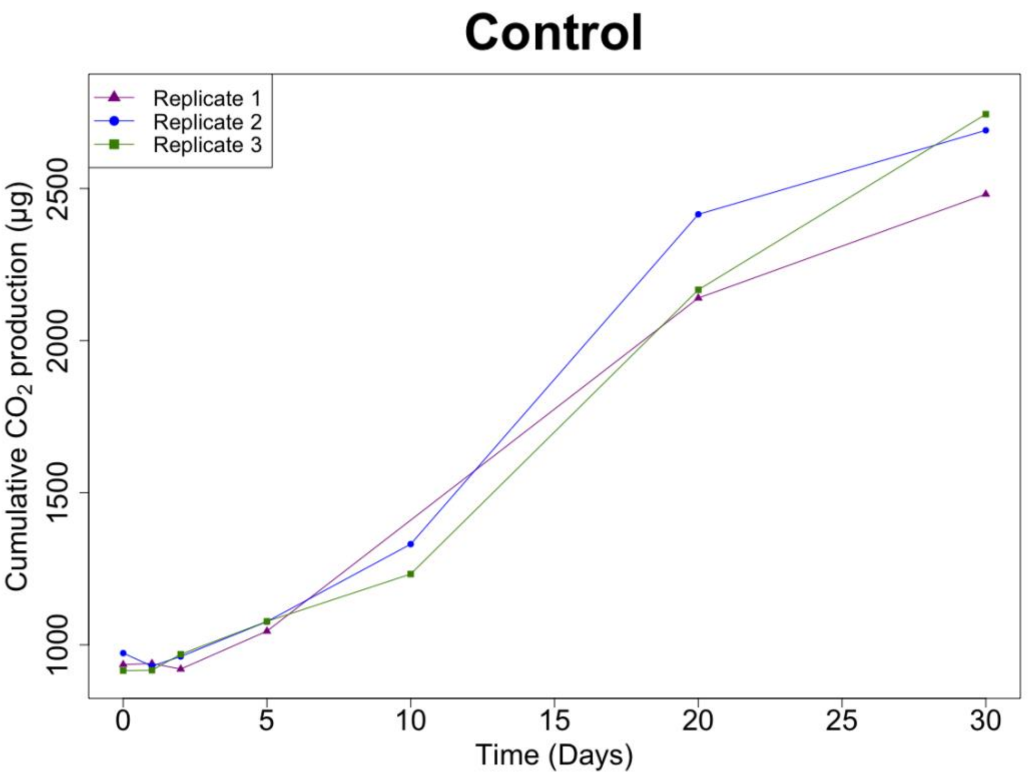

Figure 6| Cumulative CO_2 production lines for each replicate culture at 0°C with no salt in the growth medium (Control Cultures)

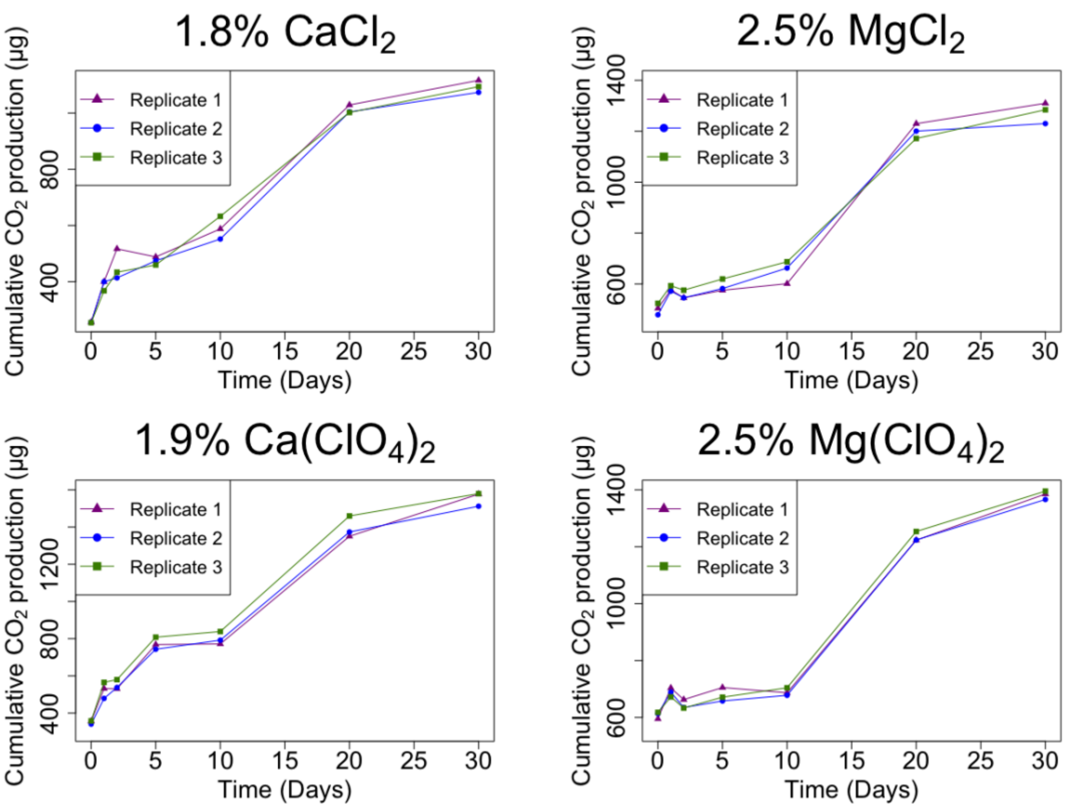

Figure 7| Cumulative CO_2 production lines for each replicate culture at 0°C with 2.5% concentration of each salt. The name of the salt type used in each case is on display in the main title on the top of each plot.

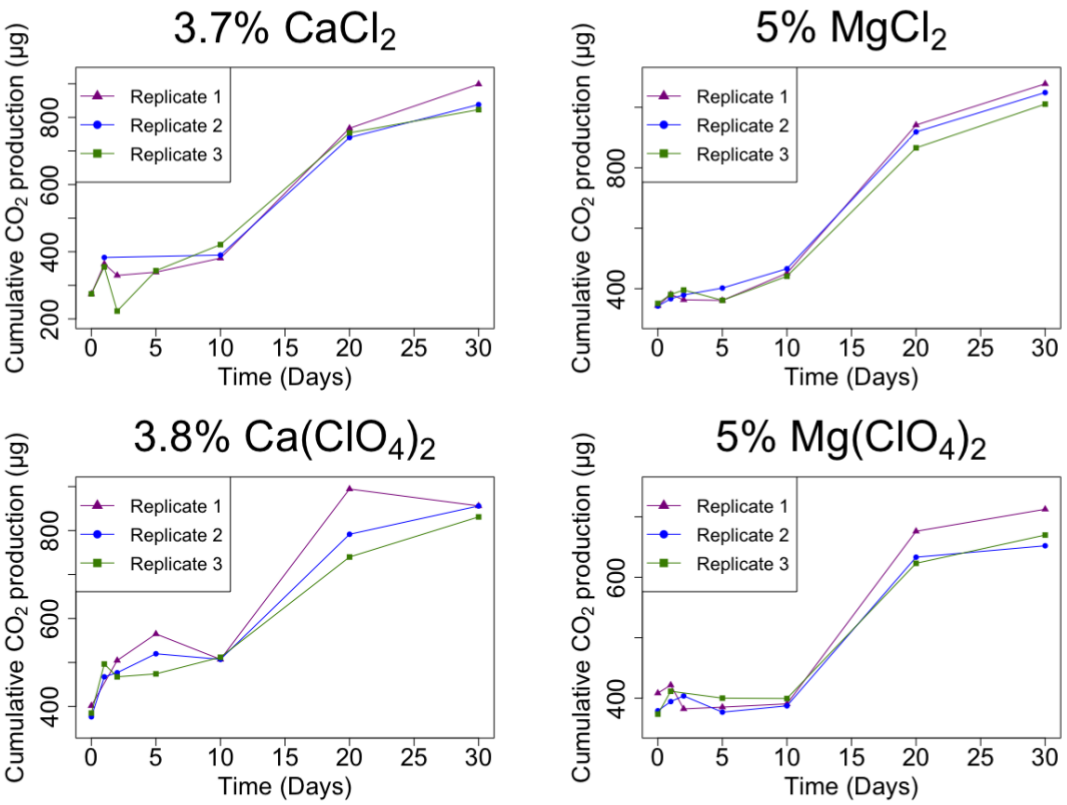

Figure 8| Cumulative CO_2 production lines for each replicate culture at 0°C with 5% concentration of each salt. The name of the salt type used in each case is on display in the main title on the top of each plot.

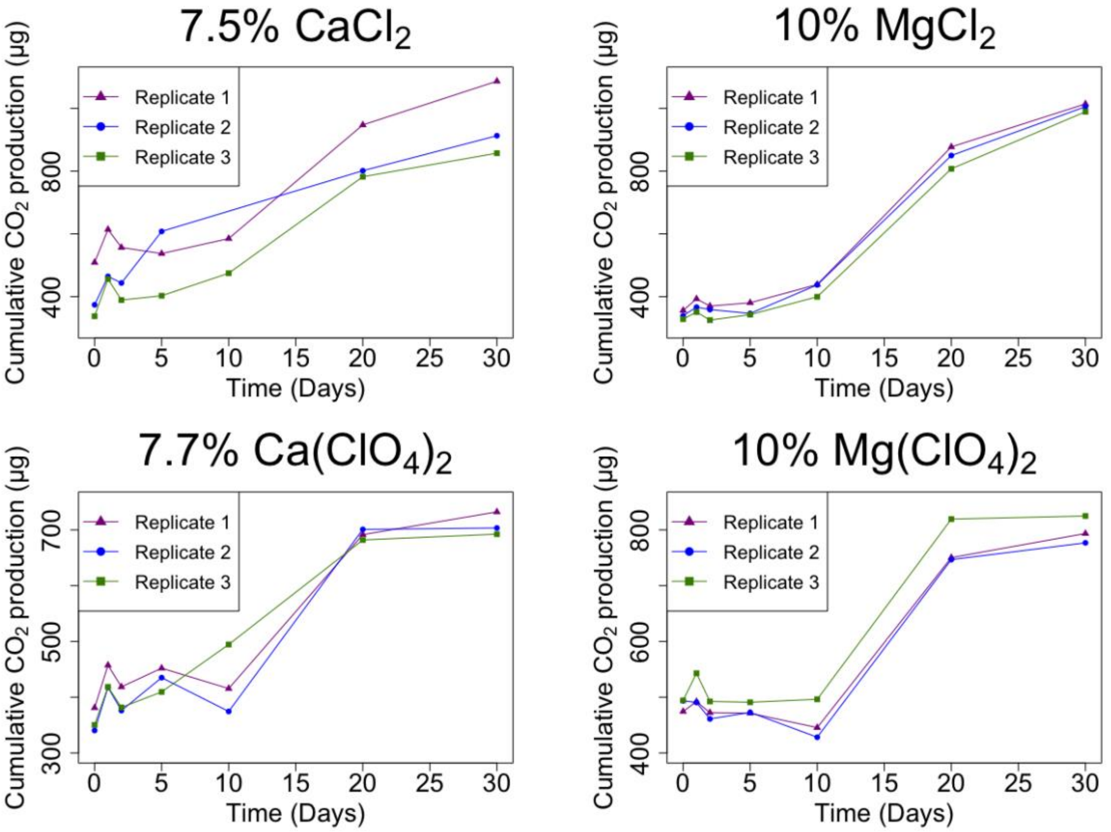

Figure 9| Cumulative CO_2 production lines for each replicate culture at 0°C with 10% concentration of each salt. The name of the salt type used in each case is on display in the main title on the top of each plot.
