# Supplementary material for: Effects of temperature, chloride and perchlorate salt concentration on the metabolic activity of Deinococcus radiodurans
Source: Extremophiles. 2024 Jul 24;28(3):34. doi: 10.1007/s00792-024-01351-5 (PMC11266278; doi:10.1007/s00792-024-01351-5)
Supplement: Supplementary file 2 — Supplementary file2 (PDF 54 KB) [file 792_2024_1351_MOESM2_ESM.pdf]

The results for the amount of the CO_2 produced in each jar seperately for each day at 25C. The lines in red were not used in the rates calculations.

Day	Treatment	weight in µg
0	CONTROL 1	797,5402091
2	CONTROL 1	2126,067908
5	CONTROL 1	4986,756582
10	CONTROL 1	10014,60468
20	CONTROL 1	16170,84557
30	CONTROL 1	19298,79835
0	CONTROL 2	833,4608734
1	CONTROL 2	1400,755304
2	CONTROL 2	2345,605385
5	CONTROL 2	15297,98202
10	CONTROL 2	17799,49555
20	CONTROL 2	24446,4714
30	CONTROL 2	24025,42933
0	CONTROL 3	788,8321693
1	CONTROL 3	1460,262113
2	CONTROL 3	2212,19032
5	CONTROL 3	5552,230384
10	CONTROL 3	9915,638807
20	CONTROL 3	17298,37799
30	CONTROL 3	20982,2002
0	1.8% CaCl2 (1)	557,7969876
1	1.8% CaCl2 (1)	648,8056168
2	1.8% CaCl2 (1)	1366,896359
5	1.8% CaCl2 (1)	2378,639936
10	1.8% CaCl2 (1)	4524,703706
20	1.8% CaCl2 (1)	7790,476453
30	1.8% CaCl2 (1)	10442,09338
0	1.8% CaCl2 (2)	210,5638994
1	1.8% CaCl2 (2)	576,2518595
2	1.8% CaCl2 (2)	1176,232292
5	1.8% CaCl2 (2)	2084,924507
10	1.8% CaCl2 (2)	4068,06054
20	1.8% CaCl2 (2)	7346,821695
30	1.8% CaCl2 (2)	9447,460429
0	1.8% CaCl2 (3)	212,1966569
1	1.8% CaCl2 (3)	612,5287381
2	1.8% CaCl2 (3)	1160,302134
5	1.8% CaCl2 (3)	2286,993085
10	1.8% CaCl2 (3)	4189,619906

20	1.8% CaCl2 (3)	7613,675915
30	1.8% CaCl2 (3)	10748,12222
0	3.8% CaCl2 (1)	242,40267
1	3.7% CaCl2 (1)	276,1720649
2	3.7% CaCl2 (1)	431,248539
5	3.7% CaCl2 (1)	519,2907956
10	3.7% CaCl2 (1)	907,1988228
20	3.7% CaCl2 (1)	1558,398092
30	3.7% CaCl2 (1)	1783,694611
0	3.7% CaCl2 (2)	149,839426
1	3.7% CaCl2 (2)	298,129123
2	3.7% CaCl2 (2)	441,9516133
5	3.7% CaCl2 (2)	512,2899944
10	3.7% CaCl2 (2)	897,970494
20	3.7% CaCl2 (2)	1550,094792
30	3.7% CaCl2 (2)	1716,273478
0	3.7% CaCl2 (3)	237,9858768
1	3.7% CaCl2 (3)	548,8850913
2	3.7% CaCl2 (3)	1085,878432
5	3.7% CaCl2 (3)	2012,052531
10	3.7% CaCl2 (3)	3662,968729
20	3.7% CaCl2 (3)	6991,701227
30	3,7% CaCl2 (3)	9613,09956
0	7.5% CaCl2 (1)	320,7226176
1	7.5% CaCl2 (1)	268,8530455
2	7.5% CaCl2 (1)	329,9426963
5	7.5% CaCl2 (1)	286,3550483
10	7.5% CaCl2 (1)	363,363861
20	7.5% CaCl2 (1)	673,4097957
30	7.5% CaCl2 (1)	769,4780718
0	7.5% CaCl2 (2)	268,5348272
1	7.5% CaCl2 (2)	317,222217
2	7.5% CaCl2 (2)	424,7769127
5	7.5% CaCl2 (2)	311,1760706
10	7.5% CaCl2 (2)	399,0043032
20	7.5% CaCl2 (2)	714,9704133
30	7.5% CaCl2 (2)	812,63533
0	7.5% CaCl2 (3)	252,3056973
1	7.5% CaCl2 (3)	319,1315264
2	7.5% CaCl2 (3)	329,1959701
5	7.5% CaCl2 (3)	323,5865817
10	7.5% CaCl2 (3)	386,9120103
20	7.5% CaCl2 (3)	747,6122919
30	7.5% CaCl2 (3)	793,8710646
0	3.5% MgCl2 (1)	457,5564582
1	2.5% MgCl2 (1)	863,2847065
2	2.5% MgCl2 (1)	1314,127714
5	2.5% MgCl2 (1)	2131,702587

10	2.5% MgCl2 (1)	3361,616061
20	2.5% MgCl2 (1)	6036,035912
30	2.5% MgCl2 (1)	7964,167721
0	2.5% MgCl2 (2)	426,6892895
1	2.5% MgCl2 (2)	798,686405
2	2.5% MgCl2 (2)	1286,498848
5	2.5% MgCl2 (2)	1966,865542
10	2.5% MgCl2 (2)	3466,30986
20	2.5% MgCl2 (2)	6394,014983
30	2.5% MgCl2 (2)	7857,439931
0	2.5% MgCl2 (3)	491,9240275
1	2.5% MgCl2 (3)	920,8822068
2	2.5% MgCl2 (3)	1519,975212
5	2.5% MgCl2 (3)	1966,547324
10	2.5% MgCl2 (3)	3173,867303
20	2.5% MgCl2 (3)	5346,777586
30	2.5% MgCl2 (3)	7095,39718
0	5% MgCl2 (1)	277,1267196
1	5% MgCl2 (1)	510,6989033
2	5% MgCl2 (1)	882,5200202
5	5% MgCl2 (1)	1335,838784
10	5% MgCl2 (1)	1982,458235
20	5% MgCl2 (1)	3446,079232
30	5% MgCl2 (1)	4358,315525
0	5% MgCl2 (2)	272,3534461
1	5% MgCl2 (2)	525,336942
2	5% MgCl2 (2)	838,960997
5	5% MgCl2 (2)	1377,207154
10	5% MgCl2 (2)	2023,508387
20	5% MgCl2 (2)	3662,983599
30	5% MgCl2 (2)	4464,752937
0	5% MgCl2 (3)	280,9453384
1	5% MgCl2 (3)	548,2486549
2	5% MgCl2 (3)	2873,04293
5	5% MgCl2 (3)	1315,472817
10	5% MgCl2 (3)	2041,965045
20	5% MgCl2 (3)	3624,650078
30	5% MgCl2 (3)	4533,523207
0	10% MgCl2 (1)	210,6191087
1	10% MgCl2 (1)	261,8522443
2	10% MgCl2 (1)	375,2440805
5	10% MgCl2 (1)	400,2771761
10	10% MgCl2 (1)	723,2686835
20	10% MgCl2 (1)	1602,119849
30	10% MgCl2 (1)	1991,961744
0	10% MgCl2 (2)	202,6636528
1	10% MgCl2 (2)	263,7615537
2	10% MgCl2 (2)	340,6457706

5	10% MgCl2 (2)	537,4292349
10	10% MgCl2 (2)	675,5359484
20	10% MgCl2 (2)	1575,089697
30	10% MgCl2 (2)	1932,769828
0	10% MgCl2 (3)	191,5260146
1	10% MgCl2 (3)	262,4886808
2	10% MgCl2 (3)	347,3663056
5	10% MgCl2 (3)	393,5945932
10	10% MgCl2 (3)	726,1326476
20	10% MgCl2 (3)	1679,92445
30	10% MgCl2 (3)	1901,440108
0	1.9% Ca(ClO4)2 (1)	240,2134044
1	1.9% Ca(ClO4)2 (1)	699,7205342
2	1.9% Ca(ClO4)2 (1)	1274,302321
5	1.9% Ca(ClO4)2 (1)	2125,974659
10	1.9% Ca(ClO4)2 (1)	3263,604845
20	1.9% Ca(ClO4)2 (1)	5750,583649
30	1.9% Ca(ClO4)2 (1)	6504,999654
0	1.9% Ca(ClO4)2 (2)	235,1219127
1	1.9% Ca(ClO4)2 (2)	713,0857001
2	1.9% Ca(ClO4)2 (2)	1296,953013
5	2.5% Ca(ClO4)2 (2)	2323,269964
10	1.9% Ca(ClO4)2 (2)	3774,663329
20	1.9% Ca(ClO4)2 (2)	6604,858656
30	1.9% Ca(ClO4)2 (2)	6441,65522
0	1.9% Ca(ClO4)2 (3)	242,7591503
1	1.9% Ca(ClO4)2 (3)	715,3132277
2	1.9% Ca(ClO4)2 (3)	1556,066975
5	1.9% Ca(ClO4)2 (3)	2314,678071
10	1.9% Ca(ClO4)2 (3)	3564,321076
20	1.9% Ca(ClO4)2 (3)	5902,5681
30	1.9% Ca(ClO4)2 (3)	6438,086309
0	3.8% Ca(ClO4)2 (1)	274,8991919
1	3.8% Ca(ClO4)2 (1)	661,2161279
2	3.8% Ca(ClO4)2 (1)	1013,19709
5	3.8% Ca(ClO4)2 (1)	1785,799367
10	3.8% Ca(ClO4)2 (1)	2110,063747
20	3.8% Ca(ClO4)2 (1)	2591,978451
30	3.8% Ca(ClO4)2 (1)	2615,468234
0	3.8% Ca(ClO4)2 (2)	223,347838
1	3.8% Ca(ClO4)2 (2)	630,6671775
2	3.8% Ca(ClO4)2 (2)	1186,935366
5	3.8% Ca(ClO4)2 (2)	2004,733512
10	3.8% Ca(ClO4)2 (2)	2279,355847
20	3.8% Ca(ClO4)2 (2)	2872,403464
30	3.8% Ca(ClO4)2 (2)	2997,363223
0	3.8% Ca(ClO4)2 (3)	336,9517475
1	3.8% Ca(ClO4)2 (3)	671,7173296

2	3.8% Ca(ClO4)2 (3)	1050,035578
5	3.8% Ca(ClO4)2 (3)	1849,124795
10	3.8% Ca(ClO4)2 (3)	2187,07256
20	3.8% Ca(ClO4)2 (3)	2727,133926
30	3.8% Ca(ClO4)2 (3)	2714,35683
0	7.7% Ca(ClO4)2 (1)	270,1259184
1	7.7% Ca(ClO4)2 (1)	315,3129076
2	7.7% Ca(ClO4)2 (1)	470,07
5	7.7% Ca(ClO4)2 (1)	330,5873829
10	7.7% Ca(ClO4)2 (1)	360,1816786
20	7.7% Ca(ClO4)2 (1)	693,6844572
30	7.7% Ca(ClO4)2 (1)	717,6712941
0	7.7% Ca(ClO4)2 (2)	265,9890814
1	7.7% Ca(ClO4)2 (2)	305,4481424
2	7.7% Ca(ClO4)2 (2)	407,6022121
5	7.7% Ca(ClO4)2 (2)	315,3129076
10	7.7% Ca(ClO4)2 (2)	388,8213197
20	7.7% Ca(ClO4)2 (2)	646,5219783
30	7.7% Ca(ClO4)2 (2)	671,7257317
0	7.7% Ca(ClO4)2 (3_	250,7146061
1	7.7% Ca(ClO4)2 (3_	270,7623549
2	7.7% Ca(ClO4)2 (3_	350,1043013
5	7.7% Ca(ClO4)2 (3_	286,6732666
10	7.7% Ca(ClO4)2 (3_	335,6788746
20	7.7% Ca(ClO4)2 (3_	603,5826892
30	7.7% Ca(ClO4)2 (3_	629,1126207
0	2.5% Mg(ClO4)2 (1)	486,1960993
1	2.5% Mg(ClO4)2 (1)	926,610135
2	2.5% Mg(ClO4)2 (1)	1425,638813
5	2.5% Mg(ClO4)2 (1)	2016,825804
10	2.5% Mg(ClO4)2 (1)	2610,621029
20	2.5% Mg(ClO4)2 (1)	3787,895706
30	2.5% Mg(ClO4)2 (1)	4561,469412
0	2.5% Mg(ClO4)2 (2)	433,0536542
1	2.5% Mg(ClO4)2 (2)	971,1606878
2	2.5% Mg(ClO4)2 (2)	1560,298423
5	2.5% Mg(ClO4)2 (2)	2310,541234
10	2.5% Mg(ClO4)2 (2)	3242,284223
20	2.5% Mg(ClO4)2 (2)	5241,731007
30	2.5% Mg(ClO4)2 (2)	5980,877138
0	2.5% Mg(ClO4)2 (3)	457,8746764
1	2.5% Mg(ClO4)2 (3)	1410,938287
2	2.5% Mg(ClO4)2 (3)	1484,381268
5	2.5% Mg(ClO4)2 (3)	1928,679354
10	2.5% Mg(ClO4)2 (3)	2616,348957
20	2.5% Mg(ClO4)2 (3)	3729,600362
30	2.5% Mg(ClO4)2 (3)	4322,25464
0	5% Mg(ClO4)2 (1)	403,4593584

1	5% Mg(ClO4)2 (1)	248,1688603
2	5% Mg(ClO4)2 (1)	316,0038088
5	5% Mg(ClO4)2 (1)	863,2847065
10	5% Mg(ClO4)2 (1)	296,8562501
20	5% Mg(ClO4)2 (1)	605,6976036
30	5% Mg(ClO4)2 (1)	599,3751351
0	5% Mg(ClO4)2 (2)	267,2619543
1	5% Mg(ClO4)2 (2)	486,8325357
2	5% Mg(ClO4)2 (2)	677,9170651
5	5% Mg(ClO4)2 (2)	798,686405
10	5% Mg(ClO4)2 (2)	960,9777043
20	5% Mg(ClO4)2 (2)	1369,505203
30	5% Mg(ClO4)2 (2)	127,9678114
0	5% Mg(ClO4)2 (3)	289,8554489
1	5% Mg(ClO4)2 (3)	491,6058092
2	5% Mg(ClO4)2 (3)	741,1398761
5	5% Mg(ClO4)2 (3)	920,8822068
10	5% Mg(ClO4)2 (3)	1039,895826
20	5% Mg(ClO4)2 (3)	1547,021012
30	5% Mg(ClO4)2 (3)	1669,846622
0	10% Mg(ClO4)2 (1)	414,2787784
1	10% Mg(ClO4)2 (1)	450,8738753
2	10% Mg(ClO4)2 (1)	540,2705516
5	10% Mg(ClO4)2 (1)	467,4212235
10	10% Mg(ClO4)2 (1)	494,7879916
20	10% Mg(ClO4)2 (1)	843,7828205
30	10% Mg(ClO4)2 (1)	915,0503968
0	10% Mg(ClO4)2 (2)	426,052853
1	10% Mg(ClO4)2 (2)	482,3774804
2	10% Mg(ClO4)2 (2)	535,5412862
5	10% Mg(ClO4)2 (2)	470,6034058
10	10% Mg(ClO4)2 (2)	544,4300361
20	10% Mg(ClO4)2 (2)	861,9789888
30	10% Mg(ClO4)2 (2)	922,7602232
0	10% Mg(ClO4)2 (3)	454,0560576
1	10% Mg(ClO4)2 (3)	451,82853
2	10% Mg(ClO4)2 (3)	551,4714433
5	10% Mg(ClO4)2 (3)	514,8357403
10	10% Mg(ClO4)2 (3)	987,3898177
20	10% Mg(ClO4)2 (3)	898,1335016
30	10% Mg(ClO4)2 (3)	997,9838794

The results for the amount of the CO_2 produced in each jar seperately for each day at 0C. The lines in red were not used in the rates calculations.

Day	Treatment	weight in µg
0	CONTROL 1	935,2164068
1	CONTROL 1	938,6295558
2	CONTROL 1	920,3226656
5	CONTROL 1	1045,057748
10	CONTROL 1	2162,132037
20	CONTROL 1	2139,862666
30	CONTROL 1	2481,328099
0	CONTROL 2	972,7610461
1	CONTROL 2	930,2518264
2	CONTROL 2	961,5907402
5	CONTROL 2	1076,396662
10	CONTROL 2	1330,996684
20	CONTROL 2	2415,078799
30	CONTROL 2	2691,15406
0	CONTROL 3	915,3580852
1	CONTROL 3	916,5992303
2	CONTROL 3	969,0376108
5	CONTROL 3	1077,017235
10	CONTROL 3	1232,674382
20	CONTROL 3	2166,880057
30	CONTROL 3	2744,207842
0	1.8% CaCl2 (1)	255,9997505
1	1.8% CaCl2 (1)	399,9725822
2	1.8% CaCl2 (1)	516,9505079
5	1.8% CaCl2 (1)	488,0938843
10	1.8% CaCl2 (1)	587,7870154
20	1.8% CaCl2 (1)	1028,806483
30	1.8% CaCl2 (1)	1116,492058
0	1.8% CaCl2 (2)	255,0688917
1	1.8% CaCl2 (2)	399,9725822
2	1.8% CaCl2 (2)	413,6251783
5	1.8% CaCl2 (2)	474,4412882
10	1.8% CaCl2 (2)	551,8444215
20	1.8% CaCl2 (2)	1004,133361
30	1.8% CaCl2 (2)	1073,733223
0	1.8% CaCl2 (3)	254,1380329
1	1.8% CaCl2 (3)	367,7028096

2	1.8% CaCl2 (3)	433,7937862
5	1.8% CaCl2 (3)	459,547547
10	1.8% CaCl2 (3)	632,6409962
20	1.8% CaCl2 (3)	1002,1606
30	1.8% CaCl2 (3)	1094,077251
0	3.7% CaCl2 (1)	274,3066408
1	3.7% CaCl2 (1)	363,669088
2	3.7% CaCl2 (1)	329,5375977
5	3.7% CaCl2 (1)	339,1564723
10	3.7% CaCl2 (1)	380,7457928
20	3.7% CaCl2 (1)	767,7111425
30	3.7% CaCl2 (1)	899,0189301
Missing measument		
1	3.7% CaCl2 (2)	382,9068371
2	3.7% CaCl2 (2)	1355,964596
5	3.7% CaCl2 (2)	8,391302974
10	3.7% CaCl2 (2)	389,954226
20	3.7% CaCl2 (2)	740,0103693
30	3.7% CaCl2 (2)	838,0239978
0	3.7% CaCl2 (3)	274,616927
1	3.7% CaCl2 (3)	354,9810723
2	3.7% CaCl2 (3)	223,4196916
5	3.7% CaCl2 (3)	343,5004801
10	3.7% CaCl2 (3)	421,1440802
20	3.7% CaCl2 (3)	753,9809819
30	3.7% CaCl2 (3)	822,9816332
0	7.5% CaCl2(1)	508,8830648
1	7.5% CaCl2(1)	614,070112
2	7.5% CaCl2(1)	556,6671511
5	7.5% CaCl2(1)	537,1191158
10	7.5% CaCl2(1)	585,1135993
20	7.5% CaCl2(1)	947,2771082
30	7.5% CaCl2(1)	1086,624955
0	7.5% CaCl2 (2)	373,9085351
1	7.5% CaCl2 (2)	464,5121274
2	7.5% CaCl2 (2)	443,4126607
5	7.5% CaCl2 (2)	607,8643865
10	7.5% CaCl2 (2)	456,1955353
20	7.5% CaCl2 (2)	801,3057294
30	7.5% CaCl2 (2)	912,8490278
0	7.5% CaCl2 (3)	337,6050409
1	7.5% CaCl2 (3)	456,134398
2	7.5% CaCl2 (3)	389,1125626
5	7.5% CaCl2 (3)	402,7651587
10	7.5% CaCl2 (3)	474,6124016
20	7.5% CaCl2 (3)	782,0789669
30	7.5% CaCl2 (3)	857,2895562
0	2.5% MgCl2 (1)	504,2287706

1	2.5% MgCl2 (1)	577,1460453
2	2.5% MgCl2 (1)	545,4968452
5	2.5% MgCl2 (1)	575,2843277
10	2.5% MgCl2 (1)	601,1540958
20	2.5% MgCl2 (1)	1229,730789
30	2.5% MgCl2 (1)	1309,725055
0	2.5% MgCl2 (2)	479,0955824
1	2.5% MgCl2 (2)	571,8711786
2	2.5% MgCl2 (2)	545,8071315
5	2.5% MgCl2 (2)	582,1106257
10	2.5% MgCl2 (2)	662,6426655
20	2.5% MgCl2 (2)	1200,491343
30	2.5% MgCl2 (2)	1230,114772
0	2.5% MgCl2 (3)	524,0870923
1	2.5% MgCl2 (3)	593,2809316
2	2.5% MgCl2 (3)	575,5946139
5	2.5% MgCl2 (3)	619,655265
10	2.5% MgCl2 (3)	687,2975026
20	2.5% MgCl2 (3)	1171,545607
30	2.5% MgCl2 (3)	1284,367934
0	5% MgCl2 (1)	343,5004801
1	5% MgCl2 (1)	380,7348331
2	5% MgCl2 (1)	363,3588017
5	5% MgCl2 (1)	361,1867978
10	5% MgCl2 (1)	451,1457494
20	5% MgCl2 (1)	941,4009103
30	5% MgCl2 (1)	1077,66442
0	5% MgCl2 (2)	342,5696213
1	5% MgCl2 (2)	366,7719508
2	5% MgCl2 (2)	379,1834018
5	5% MgCl2 (2)	402,1445861
10	5% MgCl2 (2)	465,9980609
20	5% MgCl2 (2)	918,6178859
30	5% MgCl2 (2)	1048,760496
0	5% MgCl2 (3)	351,257637
1	5% MgCl2 (3)	380,7348331
2	5% MgCl2 (3)	395,6285743
5	5% MgCl2 (3)	361,4970841
10	5% MgCl2 (3)	441,0461776
20	5% MgCl2 (3)	865,8933204
30	5% MgCl2 (3)	1010,508833
0	10% MgCl2 (1)	355,9119311
1	10% MgCl2 (1)	392,8359979
2	10% MgCl2 (1)	369,8748135
5	10% MgCl2 (1)	380,4245469
10	10% MgCl2 (1)	438,6698077
20	10% MgCl2 (1)	877,194766
30	10% MgCl2 (1)	1014,246599

0	10% MgCl2 (2)	339,1564723
1	10% MgCl2 (2)	366,4616645
2	10% MgCl2 (2)	358,7045076
5	10% MgCl2 (2)	346,2930566
10	10% MgCl2 (2)	437,4816228
20	10% MgCl2 (2)	849,7489074
30	10% MgCl2 (2)	1005,238289
0	10% MgCl2 (3)	328,2964526
1	10% MgCl2 (3)	350,9473507
2	10% MgCl2 (3)	325,1935899
5	10% MgCl2 (3)	342,5696213
10	10% MgCl2 (3)	399,4597053
20	10% MgCl2 (3)	807,3537668
30	10% MgCl2 (3)	989,2962856
0	1.9% Ca(ClO4)2 (1)	356,5325037
1	1.9% Ca(ClO4)2 (1)	531,8442491
2	1.9% Ca(ClO4)2 (1)	530,603104
5	1.9% Ca(ClO4)2 (1)	768,3911235
10	1.9% Ca(ClO4)2 (1)	771,658632
20	1.9% Ca(ClO4)2 (1)	1350,25025
30	1.9% Ca(ClO4)2 (1)	1576,89962
0	1.9% Ca(ClO4)2 (2)	340,7079036
1	1.9% Ca(ClO4)2 (2)	478,7852961
2	1.9% Ca(ClO4)2 (2)	537,4294021
5	2.5% Ca(ClO4)2 (2)	743,1421939
10	1.9% Ca(ClO4)2 (2)	791,8577757
20	1.9% Ca(ClO4)2 (2)	1373,392445
30	1.9% Ca(ClO4)2 (2)	1511,830235
0	1.9% Ca(ClO4)2 (3)	357,7736488
1	1.9% Ca(ClO4)2 (3)	565,0448806
2	1.9% Ca(ClO4)2 (3)	579,6283355
5	1.9% Ca(ClO4)2 (3)	807,8982722
10	1.9% Ca(ClO4)2 (3)	838,1969877
20	1.9% Ca(ClO4)2 (3)	1459,443796
30	1.9% Ca(ClO4)2 (3)	1579,375974
0	3.8% Ca(ClO4)2 (1)	401,5240136
1	3.8% Ca(ClO4)2 (1)	-
2	3.8% Ca(ClO4)2 (1)	504,5390569
5	3.8% Ca(ClO4)2 (1)	564,9144557
10	3.8% Ca(ClO4)2 (1)	506,9904407
20	3.8% Ca(ClO4)2 (1)	894,4839303
30	3.8% Ca(ClO4)2 (1)	856,0698865
0	3.8% Ca(ClO4)2 (2)	376,3908253
1	3.8% Ca(ClO4)2 (2)	467,3047039
2	3.8% Ca(ClO4)2 (2)	476,6132922
5	3.8% Ca(ClO4)2 (2)	519,7634287
10	3.8% Ca(ClO4)2 (2)	506,9904407
20	3.8% Ca(ClO4)2 (2)	791,5747611

30	3.8% Ca(ClO4)2 (2)	855,9465075
0	3.8% Ca(ClO4)2 (3)	384,7685547
1	3.8% Ca(ClO4)2 (3)	496,4716138
2	3.8% Ca(ClO4)2 (3)	467,3047039
5	3.8% Ca(ClO4)2 (3)	474,0183092
10	3.8% Ca(ClO4)2 (3)	511,7431804
20	3.8% Ca(ClO4)2 (3)	739,8621089
30	3.8% Ca(ClO4)2 (3)	831,0541139
0	7.7% Ca(ClO4)2 (1)	381,0451194
1	7.7% Ca(ClO4)2 (1)	457,3755431
2	7.7% Ca(ClO4)2 (1)	418,5897587
5	7.7% Ca(ClO4)2 (1)	452,0368881
10	7.7% Ca(ClO4)2 (1)	415,5002018
20	7.7% Ca(ClO4)2 (1)	691,4865837
30	7.7% Ca(ClO4)2 (1)	732,2448966
0	7.7% Ca(ClO4)2 (2)	340,3976174
1	7.7% Ca(ClO4)2 (2)	417,6588999
2	7.7% Ca(ClO4)2 (2)	376,080539
5	7.7% Ca(ClO4)2 (2)	435,105253
10	7.7% Ca(ClO4)2 (2)	374,507822
20	7.7% Ca(ClO4)2 (2)	700,8781913
30	7.7% Ca(ClO4)2 (2)	703,4315877
0	7.7% Ca(ClO4)2 (3_	350,3267782
1	7.7% Ca(ClO4)2 (3_	418,5897587
2	7.7% Ca(ClO4)2 (3_	381,665692
5	7.7% Ca(ClO4)2 (3_	409,5592772
10	7.7% Ca(ClO4)2 (3_	494,5144991
20	7.7% Ca(ClO4)2 (3_	681,9742704
30	7.7% Ca(ClO4)2 (3_	692,3652476
0	2.5% Mg(ClO4)2 (1)	595,1426493
1	2.5% Mg(ClO4)2 (1)	703,4325593
2	2.5% Mg(ClO4)2 (1)	662,7850572
5	2.5% Mg(ClO4)2 (1)	705,1202764
10	2.5% Mg(ClO4)2 (1)	686,7034101
20	2.5% Mg(ClO4)2 (1)	1223,137553
30	2.5% Mg(ClO4)2 (1)	1385,230931
0	2.5% Mg(ClO4)2 (2)	613,4495395
1	2.5% Mg(ClO4)2 (2)	688,8491044
2	2.5% Mg(ClO4)2 (2)	634,8592925
5	2.5% Mg(ClO4)2 (2)	657,8899258
10	2.5% Mg(ClO4)2 (2)	677,7920232
20	2.5% Mg(ClO4)2 (2)	1223,676171
30	2.5% Mg(ClO4)2 (2)	1366,02014
0	2.5% Mg(ClO4)2 (3)	617,7935473
1	2.5% Mg(ClO4)2 (3)	672,0936455
2	2.5% Mg(ClO4)2 (3)	632,9975748
5	2.5% Mg(ClO4)2 (3)	671,2570062
10	2.5% Mg(ClO4)2 (3)	704,2291377

20	2.5% Mg(ClO4)2 (3)	1253,491797
30	2.5% Mg(ClO4)2 (3)	1395,390093
0	5% Mg(ClO4)2 (1)	408,3503116
1	5% Mg(ClO4)2 (1)	421,6926215
2	5% Mg(ClO4)2 (1)	382,2862645
5	5% Mg(ClO4)2 (1)	385,2014863
10	5% Mg(ClO4)2 (1)	390,5483184
20	5% Mg(ClO4)2 (1)	676,1206235
30	5% Mg(ClO4)2 (1)	712,522836
0	5% Mg(ClO4)2 (2)	379,1834018
1	5% Mg(ClO4)2 (2)	394,3874292
2	5% Mg(ClO4)2 (2)	403,6960175
5	5% Mg(ClO4)2 (2)	376,8841918
10	5% Mg(ClO4)2 (2)	387,2808099
20	5% Mg(ClO4)2 (2)	633,2338862
30	5% Mg(ClO4)2 (2)	652,1094984
0	5% Mg(ClO4)2 (3)	373,5982488
1	5% Mg(ClO4)2 (3)	411,4531744
2	5% Mg(ClO4)2 (3)	-
5	5% Mg(ClO4)2 (3)	400,0537978
10	5% Mg(ClO4)2 (3)	399,4597053
20	5% Mg(ClO4)2 (3)	623,1104357
30	5% Mg(ClO4)2 (3)	669,8174658
0	10% Mg(ClO4)2 (1)	474,4412882
1	10% Mg(ClO4)2 (1)	492,1276059
2	10% Mg(ClO4)2 (1)	472,2692843
5	10% Mg(ClO4)2 (1)	471,3448931
10	10% Mg(ClO4)2 (1)	445,501871
20	10% Mg(ClO4)2 (1)	750,3423303
30	10% Mg(ClO4)2 (1)	793,4760997
0	10% Mg(ClO4)2 (2)	493,6790373
1	10% Mg(ClO4)2 (2)	490,5761745
2	10% Mg(ClO4)2 (2)	461,0989784
5	10% Mg(ClO4)2 (2)	472,8301242
10	10% Mg(ClO4)2 (2)	428,2731897
20	10% Mg(ClO4)2 (2)	746,736237
30	10% Mg(ClO4)2 (2)	776,8126724
0	10% Mg(ClO4)2 (3)	494,2996098
1	10% Mg(ClO4)2 (3)	543,014555
2	10% Mg(ClO4)2 (3)	492,4378922
5	10% Mg(ClO4)2 (3)	490,9499443
10	10% Mg(ClO4)2 (3)	496,2967764
20	10% Mg(ClO4)2 (3)	819,3712216
30	10% Mg(ClO4)2 (3)	824,9269083
