# Supplementary material for: Effects of temperature, chloride and perchlorate salt concentration on the metabolic activity of Deinococcus radiodurans
Source: Extremophiles. 2024 Jul 24;28(3):34. doi: 10.1007/s00792-024-01351-5 (PMC11266278; doi:10.1007/s00792-024-01351-5)
Supplement: Supplementary file 3 — Supplementary file3 (PDF 51 KB) [file 792_2024_1351_MOESM3_ESM.pdf]

The calculations of the average CO_2 production rates was made with linear regression analysis of the increase in CO_2 concentration over the first twenty days of the incubation. The values in red were not used in the calculation of the average rate. The rates in Figure 1 of the main manuscript are presented in units of µg/day.

Sample	Tempearure (℃)	Intercept	Day Range	Df	Rate (mg/day)	Standard Error for linear fit	R squared for linear fit	p-values for linear fit	Average Rate for all samples of same tratment (mg/day)	Standard deviation from average rate	Standard error from average rate
Control 1	0	0,841	0 - 20	5	0,063	0,093	0,977	0,002			
Control 2	0	0,818	0 - 20	5	0,073	0,160	0,938	0,001	0,0660	0,0062	0,00359
Control 3	0	0,823	0 - 20	5	0,062	0,130	0,942	0,001			
1.8% CaCl2 1	0	0,337	0 - 20	5	0,033	0,084	0,919	0,003			
1.8% CaCl2 2	0	0,307	0 - 20	5	0,033	0,064	0,950	0,001	0,0335	0,0008	0,00044
1.8% CaCl2 3	0	0,307	0 - 20	5	0,034	0,044	0,978	0,000			
3.7% CaCl2 1	0	0,271	0 - 20	5	0,022	0,075	0,861	0,008			
3.7% CaCl2 2	0	0,307	0 - 20	3	0,019	0,132	0,790	0,303	0,0215	0,0022	0,00128
3.7% CaCl2 3	0	0,247	0 - 20	5	0,023	0,068	0,897	0,004			
7.5% CaCl2 1	0	0,443	2 - 20	3	0,023	0,086	0,871	0,067			
7.5% CaCl2 2	0	0,429	0 - 20	4	0,020	0,060	0,906	0,013	0,0207	0,0020	0,00117
7.5% CaCl2 3	0	0,350	0 - 20	5	0,019	0,064	0,870	0,007			
2.5% MgCl2 1	0	0,378	2 - 20	3	0,039	0,143	0,873	0,065			
2.5% MgCl2 2	0	0,402	2 - 20	3	0,037	0,101	0,928	0,037	0,0367	0,0027	0,00153
2.5% MgCl2 3	0	0,451	2 - 20	3	0,034	0,088	0,932	0,035			
5% MgCl2 1	0	0,218	2 - 20	3	0,034	0,100	0,914	0,044			
5% MgCl2 2	0	0,256	2 - 20	3	0,031	0,088	0,919	0,041	0,0309	0,0028	0,00163
5% MgCl2 3	0	0,257	2 - 20	3	0,028	0,099	0,882	0,061			
10% MgCl2 1	0	0,246	2 - 20	3	0,029	0,091	0,906	0,048			
10% MgCl2 2	0	0,231	2 - 20	3	0,029	0,084	0,916	0,043	0,0286	0,0008	0,00048
10% MgCl2 3	0	0,213	2 - 20	3	0,028	0,081	0,916	0,043			
1.9% Ca(ClO4)2 1	0	0,437	0 - 20	5	0,044	0,092	0,945	0,001			
1.9% Ca(ClO4)2 2	0	0,412	0 - 20	5	0,047	0,078	0,964	0,001	0,0468	0,0022	0,00129
1.9% Ca(ClO4)2 3	0	0,458	0 - 20	5	0,049	0,097	0,949	0,001			
3.8% Ca(ClO4)2 1	0	0,414	0 - 20	4	0,022	0,085	0,847	0,027			
3.8% Ca(ClO4)2 2	0	0,413	0 - 20	5	0,017	0,053	0,886	0,005	0,0179	0,0035	0,00204
3.8% Ca(ClO4)2 3	0	0,419	0 - 20	5	0,015	0,049	0,868	0,007			
7.7% Ca(ClO4)2 1	0	0,356	2 - 20	3	0,015	0,074	0,792	0,110			
7.7% Ca(ClO4)2 2	0	0,311	2 - 20	3	0,017	0,090	0,776	0,119	0,0159	0,0013	0,00073
7.7% Ca(ClO4)2 3	0	0,358	0 - 20	5	0,015	0,029	0,954	0,001			
2.5% Mg(ClO4)2 1	0	0,529	2 - 20	3	0,031	0,131	0,843	0,082			
2.5% Mg(ClO4)2 2	0	0,488	2 - 20	3	0,034	0,126	0,869	0,068	0,0334	0,0019	0,00109
2.5% Mg(ClO4)2 3	0	0,491	2 - 20	3	0,035	0,118	0,892	0,055			
5% Mg(ClO4)2 1	0	0,302	2 - 20	3	0,017	0,070	0,843	0,082			
5% Mg(ClO4)2 2	0	0,323	2 - 20	3	0,014	0,069	0,785	0,114	0,0141	0,0026	0,00151
5% Mg(ClO4)2 3	0	0,353	1 - 20	3	0,012	0,066	0,763	0,127			
10% Mg(ClO4)2 1	0	0,388	2 - 20	3	0,016	0,087	0,759	0,129			
10% Mg(ClO4)2 2	0	0,379	2 - 20	3	0,016	0,093	0,736	0,142	0,0169	0,0017	0,00096
10% Mg(ClO4)2 3	0	0,400	2 - 20	3	0,019	0,082	0,833	0,087			
Control 1	25	1,007	0 - 20	5	0,785	0,791	0,988	0,001			
Control 2	25	2,560	0 - 20	5	1,231	4,337	0,853	0,008	0,8126	0,0385	0,0272116
Control 3	25	0,886	0 - 20	5	0,840	0,492	0,995	0,000			
1.8% CaCl2 1	25	0,536	0 - 20	5	0,370	0,212	0,995	0,000			
1.8% CaCl2 2	25	0,322	0 - 20	5	0,356	0,147	0,998	0,000	0,3649	0,0076	0,0025389
1.8% CaCl2 3	25	0,343	0 - 20	5	0,369	0,141	0,998	0,000			
3.7% CaCl2 1	25	0,239	0 - 20	5	0,066	0,042	0,994	0,000			
3.7% CaCl2 2	25	0,218	0 - 20	5	0,067	0,061	0,989	0,000	0,0664	0,0007	0,0004921
3.7% CaCl2 3	25	0,296	0 - 20	5	0,336	0,081	0,999	0,000			
7.5% CaCl2 1	25	0,259	0 - 20	5	0,018	0,067	0,844	0,010			
7.5% CaCl2 2	25	0,284	0 - 20	5	0,019	0,077	0,819	0,013	0,0200	0,0022	0,0012592
7.5% CaCl2 3	25	0,251	0 - 20	5	0,022	0,061	0,907	0,003			
2.5% MgCl2 1	25	0,636	0 - 20	5	0,272	0,134	0,997	0,000			
2.5% MgCl2 2	25	0,531	0 - 20	5	0,294	0,101	0,998	0,000	0,2663	0,0306	0,0176717
2.5% MgCl2 3	25	0,760	0 - 20	5	0,233	0,211	0,989	0,000			
5% MgCl2 1	25	0,436	0 - 20	5	0,153	0,134	0,989	0,000			
5% MgCl2 2	25	0,411	0 - 20	5	0,164	0,117	0,993	0,000	0,1604	0,0064	0,0036956
5% MgCl2 3	25	0,380	0 - 20	4	0,164	0,091	0,997	0,000			
10% MgCl2 1	25	0,165	0 - 20	5	0,068	0,100	0,971	0,000			
10% MgCl2 2	25	0,181	0 - 20	5	0,066	0,094	0,973	0,000	0,0688	0,0033	0,0019082
10% MgCl2 3	25	0,141	0 - 20	5	0,073	0,110	0,969	0,000			
1.9% Ca(ClO4)2 1	25	0,541	0 - 20	5	0,266	0,236	0,989	0,000			
1.9% Ca(ClO4)2 2	25	0,519	0 - 20	5	0,311	0,234	0,992	0,000	0,2830	0,0248	0,0143123
1.9% Ca(ClO4)2 3	25	0,663	0 - 20	5	0,272	0,355	0,977	0,000			
3.8% Ca(ClO4)2 1	25	0,725	0 - 20	5	0,108	0,415	0,830	0,012			

3.8% Ca(ClO4)2 2	25	0,766	0 - 20	5	0,121	0,496	0,812	0,014	0,1137	0,0068	0,0039336
3.8% Ca(ClO4)2 3	25	0,758	0 - 20	5	0,113	0,410	0,845	0,010			
7.7% Ca(ClO4)2 1	25	0,252	0 - 20	4	0,020	0,065	0,890	0,016			
7.7% Ca(ClO4)2 2	25	0,253	0 - 20	4	0,018	0,042	0,945	0,006	0,0183	0,0013	0,0007695
7.7% Ca(ClO4)2 3	25	0,227	0 - 20	4	0,017	0,048	0,917	0,010			
2.5% Mg(ClO4)2 1	25	0,903	0 - 20	5	0,154	0,324	0,942	0,001			
2.5% Mg(ClO4)2 2	25	0,854	0 - 20	5	0,227	0,314	0,974	0,000	0,1744	0,0462	0,026666
2.5% Mg(ClO4)2 3	25	1,038	0 - 20	5	0,142	0,352	0,922	0,002			
5% Mg(ClO4)2 1	25	0,291	0 - 20	4	0,013	0,107	0,572	0,139			
5% Mg(ClO4)2 2	25	0,455	0 - 20	5	0,048	0,128	0,912	0,003	0,0519	0,0052	0,0036463
5% Mg(ClO4)2 3	25	0,487	0 - 20	5	0,056	0,149	0,909	0,003			
10% Mg(ClO4)2 1	25	0,389	0 - 20	4	0,020	0,070	0,880	0,018			
10% Mg(ClO4)2 2	25	0,432	0 - 20	5	0,019	0,065	0,863	0,007	0,0218	0,0037	0,0021351
10% Mg(ClO4)2 3	25	0,478	0 - 20	5	0,026	0,145	0,699	0,038			
